# High Young’s modulus NaF coating on bulk Sn: mechanical confinement effect drives durable sodium storage by alleviating (de)sodiation stress

**DOI:** 10.1093/nsr/nwag428

**Published:** 2026-07-11

**Authors:** Huiqian Xiong, Benchi Li, Jianing Wang, Yanxia Wang, Zhaoyu Rong, Yakun Tang, Yue Zhang, Yongjin Fang, Zhongxue Chen, Jianyu Huang, Yuliang Cao

**Affiliations:** College of Chemistry and Molecular Sciences, Hubei Key Laboratory of Electrochemical Power Sources, Wuhan University, Wuhan 430072, China; College of Chemistry and Molecular Sciences, Hubei Key Laboratory of Electrochemical Power Sources, Wuhan University, Wuhan 430072, China; College of Chemistry and Molecular Sciences, Hubei Key Laboratory of Electrochemical Power Sources, Wuhan University, Wuhan 430072, China; College of Chemistry and Molecular Sciences, Hubei Key Laboratory of Electrochemical Power Sources, Wuhan University, Wuhan 430072, China; Clean Nano Energy Center, State Key Laboratory of Metastable Materials Science and Technology, Yanshan University, Qinhuangdao 066004, China; State Key Laboratory of Chemistry and Utilization of Carbon-based Energy Resources; College of Chemistry, Xinjiang University, Urumqi 830017, China; State Key Laboratory of Chemistry and Utilization of Carbon-based Energy Resources; College of Chemistry, Xinjiang University, Urumqi 830017, China; College of Chemistry and Molecular Sciences, Hubei Key Laboratory of Electrochemical Power Sources, Wuhan University, Wuhan 430072, China; School of Power and Mechanical Engineering, Ministry of Education, Wuhan University, Wuhan 430072, China; Clean Nano Energy Center, State Key Laboratory of Metastable Materials Science and Technology, Yanshan University, Qinhuangdao 066004, China; College of Chemistry and Molecular Sciences, Hubei Key Laboratory of Electrochemical Power Sources, Wuhan University, Wuhan 430072, China; State Key Laboratory of Chemistry and Utilization of Carbon-based Energy Resources; College of Chemistry, Xinjiang University, Urumqi 830017, China; Perception and Effectiveness Assessment for Carbon-neutrality Efforts, Engineering Research Center of Ministry of Education, Institute for Carbon Neutrality, Wuhan University, Wuhan 430072, China

**Keywords:** sodium-ion batteries, anode materials, bulk Sn, stress buffer, high modulus

## Abstract

Sn has emerged as a promising anode material for next-generation sodium-ion batteries (SIBs) owing to its high theoretical capacity. However, the high volumetric expansion of Sn anode upon sodiation generates significant internal stress, which leads to particle cracking, pulverization and electrode failure. Herein, we report a novel and facile approach to *in situ* coat a sodium fluoride (NaF) layer on bulk Sn particles via a solution supersaturation route. The high Young’s modulus of the NaF coating enables a strong mechanical confinement effect, which effectively suppresses the volume expansion and mechanical stress of Sn. Additionally, the NaF coating promotes the formation of a thin, uniform and dense solid electrolyte interphase film, thereby resulting in a significantly improved Coulombic efficiency. The designed Sn@NaF anode exhibits remarkable cycling stability and low-temperature adaptability in both half cell and full cell. These results highlight the great potential of the Sn@NaF anode for high-energy-density SIBs.

## Teaser

An NaF surface layer helps bulk Sn withstand the strain of repeated sodium storage and release, markedly extending battery lifetime while maintaining high-energy-storage performance.

## INTRODUCTION

The growing scarcity of lithium resources and their surging prices have propelled sodium-ion batteries (SIBs) into the spotlight [1–5]. Sharing the same working principle as lithium-ion batteries (LIBs), SIBs have emerged as an ideal option for large-scale energy-storage applications [6–8]. The anode material is a critical component of SIBs, which significantly influences the operating voltage and energy density of the battery [6,9–13]. However, the larger ionic radius of sodium ions (1.02 Å) compared to lithium ions (0.76 Å) prevents the direct employment of anode materials that are well-established in LIBs [5,8]. It is well recognized that the Na^+^ storage performance of graphite in SIBs is significantly hindered by the insufficient interlayer spacing and thermodynamic instability of the binary Na-intercalated graphite compound. As another carbonaceous material, hard carbon (HC) is capable of repeatably storing/releasing Na^+^, thereby receiving considerable attention as the most promising anode material for commercial applications. However, the unsatisfactory reversible capacity and low tap density of HC result in uncompetitive energy density. Besides, the low operating voltage close to that of the sodium metal plating would induce serious safety concerns [14–17].

Sn-based materials are highly anticipated as promising anodes for high-energy-density SIBs due to the high theoretical capacity (847 mAh g^–1^ and 6150 mAh cm^–3^) and appropriate average operating voltage (∼0.3 V vs. Na^+^/Na) [18–27]. Nevertheless, Sn suffers from a massive volume expansion of 420% during the Na_15_Sn_4_ alloying process [18–27], which leads to particle breakage and pulverization upon cycling [28–33]. Finally, the active particles lose electrical contact with the current collector, resulting in a significant decrease in capacity [34–37]. Recent works have shown that reducing the size of bulk Sn to the nanometer scale can effectively decrease mechanical stress and avoid fracture [37,38]. Therefore, a variety of Sn nanoarchitectures, such as nanodots, nanofibers, nanospheres, have been extensively investigated [39,40,30,41,42]. Despite the greatly improved electrochemical performance, nanoparticle engineering brings other insurmountable issues, including reduced packing density and low initial Coulombic efficiency (ICE) induced by the excessive formation of solid electrolyte interphase (SEI) film. Thus, developing high-performance microsized Sn anodes is highly desirable for commercial applications. Nevertheless, Amine and co-workers have revealed that microsized Sn was pulverized into nanoparticles with simultaneous formation of numerous voids and pores owing to the large mechanical stress upon the first charge/discharge [43]. As such, constructing a confined layer on the surface of micron Sn to alleviate the mechanical stress will be a feasible route to improve its sodium storage performance.

Theoretically, the confinement agent should possess a high modulus, so that it could endure continuously repeated mechanical strain-release processes during the sodiation/desodiation of Sn anode. Ideally, the confinement agent can serve as one of the components of the SEI film, thereby increasing the mechanical strength of the SEI film and reducing the irreversible capacity loss caused by the formation of the SEI film [44–47]. Sodium fluoride (NaF) is a well-documented inorganic component of the SEI films in SIBs. Compared with other inorganic (Na_2_CO_3_, Na_2_O, Na_2_SO_4_, etc.) and organic (RONa, ROCO_2_Na, RCOONa, etc.; R denotes an alkyl group) components, NaF has the highest Young’s modulus (76 GPa), and hence can enhance the mechanical properties of the SEI film. On this account, many previous works have been devoted to increasing the content of NaF in the SEI film by electrolyte engineering. Unfortunately, the content of NaF in the SEI is insufficient to buffer the volume expansion of alloying (Sn, Sb, P, etc.) anodes due to the inevitable formation of other inorganic and organic components meanwhile.

Herein, we developed a novel strategy for *in situ* coating of NaF on the surface of bulk Sn (Sn@NaF) based on the supersaturation theory. The high Young’s modulus of the NaF coating creates a high hoop stress, which effectively regulates the Na^+^ flux, and suppresses the volume expansion and mechanical stress of Sn during (de)sodiation. Benefiting from the strong mechanical confinement effect of NaF coating, the Sn@NaF anode exhibits excellent cycling stability (98% capacity retention after 200 cycles at 1C, 81% capacity retention after 4500 cycles at 5C). In addition, the coating NaF participates in the formation of a thin and dense SEI layer, thereby reducing the side reactions, and enabling a high ICE and energy conversion efficiency. More significantly, the Sn@NaF||Na_3_V_2_(PO_4_)_3_ (NVP) coin full cell also manifests excellent long-term cycling stability (capacity retention of 83% after 500 cycles at 5C) and low-temperature adaptability (a capacity of 309.6 mAh g^−1^ after 900 cycles at −40°C). More significantly, the assembled Sn@NaF||NVP pouch cell also demonstrates a high ICE of 87% and a stable cycle life during 100 cycles, which fully signifies the practical application potential of the Sn@NaF anode.

## RESULTS AND DISCUSSION

Initially, Sn particles were dispersed uniformly in an aqueous NaF solution to form a homogeneous suspension. Through a controlled slow-drying process, the concentration of NaF was gradually increased to a supersaturated state. This induced the preferential growth of a coating layer at the surface defects of the Sn particles, which served as nucleation sites. The coating subsequently propagated along branch-like crystal growth directions, ultimately leading to the complete encapsulation of the Sn particles. As a result, an *in situ* and uniform NaF coating was achieved on the bulk Sn surface.

Figure 1a, b and Fig. S1 show the scanning electron microscopy (SEM) images of uncoated Sn (denoted as Bare Sn) and Sn@NaF. Bare Sn particles exhibit spherical shape and smooth surface, with diameters ranging from 2 to 30 μm. The surface of Sn@NaF particles becomes significantly rougher (Fig. 1b), implying the existence of the NaF coating layer. Therefore, the uniformity and controllability of the coating layer are critically important to affect the electrochemical performance of the bulk Sn anode. The NaF coating on bulk Sn was synthesized based on the theory of supersaturated solution, and the growth process of the NaF coating is visually conveyed by the SEM images in Fig. S2. When NaF concentration in the dispersion reached a supersaturated state through a precisely controlled slow drying process, it triggered the preferential growth of the coating layer at the surface defects of the Sn particles (Fig. S2a), where the defects act as nucleation centers. The coating layer propagates along the ‘branch-like’ crystal growth directions (Fig. S2b) until the complete encapsulation of Sn particles. As such, the *in situ* and uniform NaF coating on the surface of bulk Sn is achieved. Brunauer–Emmett–Teller surface area analysis further proves the morphological transformation. As depicted in Fig. S3, the specific surface area of the Sn anode experiences a remarkable increase after NaF coating, escalating from the initial 0.06 to 0.7 m^2^ g^–1^, which can be directly attributed to the enhanced surface roughness. High-angle annular dark-field scanning transmission electron microscopy (HAADF-STEM) imaging (Fig. 1c) accurately characterizes the coating layer, and determines its thickness in the range of 80–100 nm. Energy-dispersive X-ray spectroscopy (EDS) analysis clearly indicates the uniformly distributed NaF coating layer on the surface of Sn. EDS mapping (Fig. S4a–d) further corroborates the complete coverage of Sn particles by the NaF coating. Notably, the absence of lattice fringes in Fig. S5 indicates that the coated NaF is amorphous. The disordered atomic arrangement and flexible structure of amorphous NaF endow it with excellent durability to the volume expansion of bulk Sn. Through structural relaxation, the amorphous NaF coating effectively mitigates interfacial stress, thereby reducing the likelihood of coating layer cracking during cycling. X-ray diffraction (XRD) measurement in Fig. S6 implies that the NaF coating does not affect the crystal structure of bulk Sn. Note that no characteristic XRD peak of NaF is detected, further confirming the amorphous state of the coated NaF.

**Figure 1.**
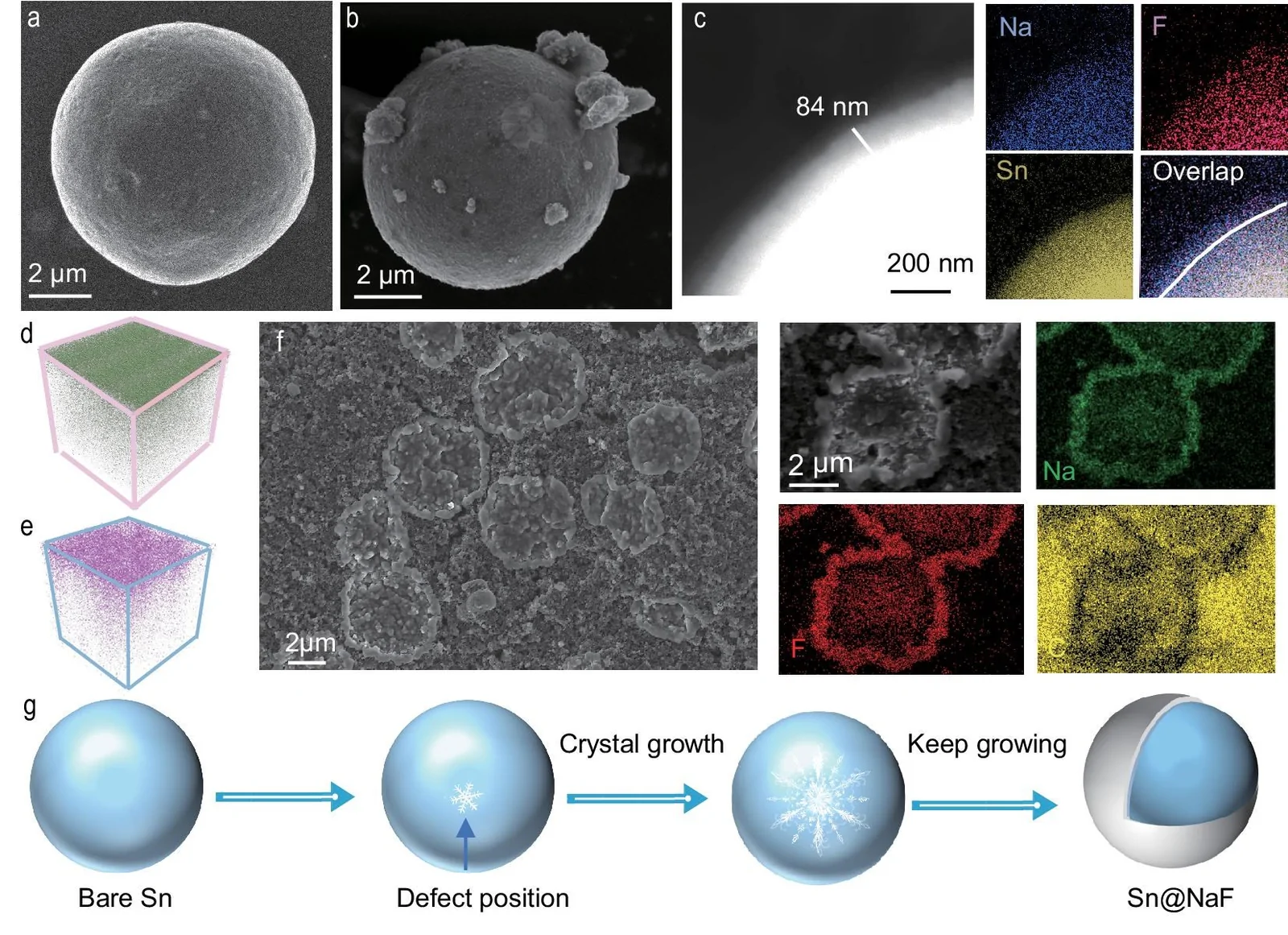
Morphological and structural characterization of Sn@NaF and Bare Sn. (a and b) SEM images of Bare Sn and Sn@NaF. (c) HAADF-STEM image and the corresponding elemental mappings. (d and e) TOF-SIMS of F^–^ and Na^+^. (f) SEM image of the electrode made of Sn@NaF and the corresponding elemental mappings. (g) Schematic diagram of the coating process of NaF.

Time-of-flight secondary ion mass spectrometry (TOF-SIMS) analysis (Fig. 1d and e) shows that F^−^ and Na⁺ are exclusively concentrated on the surface in the form of an NaF coating layer. The depth-distribution curves of F^−^ and Na⁺ as a function of sputtering time, presented in Fig. S7, further confirm the uniform distribution of the NaF coating layer. SEM image of the Sn@NaF electrode sheet (Fig. 1f) reveals a distinctive cell-wall-like structure. In this structure, NaF uniformly encapsulates individual Sn particles, acting as a confining framework that only permits microscopic volume expansion due to lattice changes, while avoiding the macroscopic volume expansion caused by fragmentation and pulverization of Sn particles. This confinement significantly suppresses material fragmentation and pulverization, which is favorable to the electrochemical performance of the anode. EDS characterization further elucidates the composition of this cell wall structure. The analysis confirms that the cell wall is primarily composed of NaF, and due to the complete coating, no obvious signal of Sn was observed (Fig. S8). The growth process of the NaF coating on Sn crystals is schematically illustrated in Fig. 1g.

Phase characterizations strongly prove the successful coating of NaF. In order to test the electrochemical influence of the coating layer on bulk Sn, the electrochemical performances of Sn@NaF and Bare Sn were comparatively evaluated. As depicted in Fig. 2a, both samples exhibit multiple discharge/charge voltage plateaus, indicative of the multi-step alloying/dealloying reaction between Sn and Na. Sn@NaF delivers a charge capacity of 739.9 mAh g^–1^ with an ICE of 84% at 0.1C (1C = 847 mA g^–1^), both of which are much higher than those of Bare Sn (654.3 mAh g^–1^ and 77%) and other Sn-based anodes (Fig. S9). The nearly overlapped charge–discharge curves of Sn@NaF underscore its high electrochemical reversibility (Fig. 2b). In contrast, the capacity of Bare Sn declines sharply from 602 to 405 mAh g^–1^ in 200 cycles, while the plateau capacity gradually diminishes along cycling (Fig. 2c). Due to the fragmentation and pulverization of the particles, there was a rapid decline afterward. The cycling performances in Fig. 2d show that Sn@NaF retains a capacity retention of 98% after 200 cycles. Before the cycle at 1C, the batteries were activated two cycles at current densities of 0.2C and 0.5C, respectively. A detailed comparison of the cyclic Coulombic efficiency (CCE) during cycling reveals that Sn@NaF maintains an average CCE of 99.9% over 200 cycles, higher than that (99.58%) of Bare Sn.

**Figure 2.**
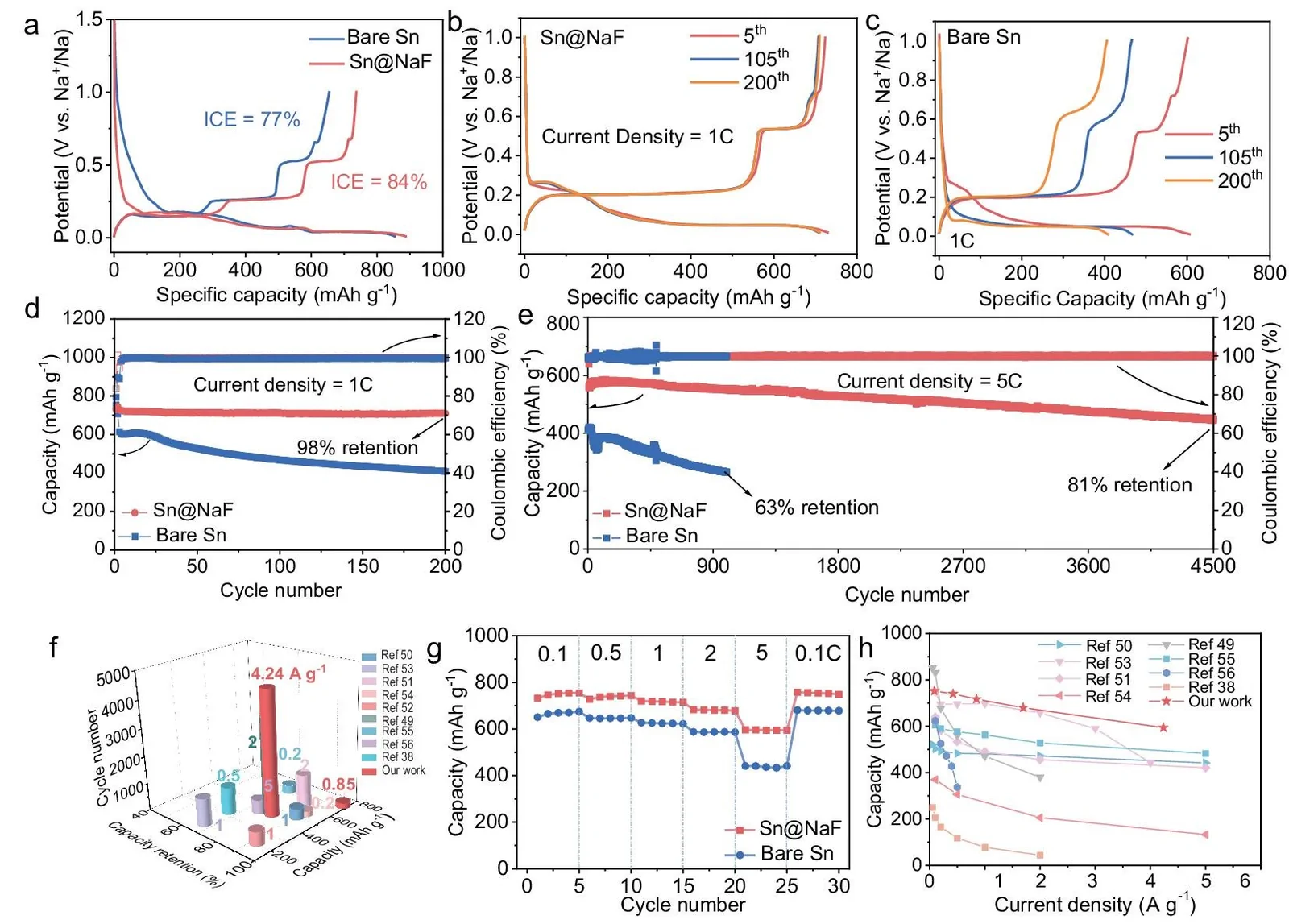
Electrochemical performance of Sn@NaF and Bare Sn. (a) Initial charge–discharge curves at 0.1C. (b and c) Galvanostatic discharge/charge profiles of Sn@NaF and Bare Sn at 1C. (d) Cycling performance of Sn@NaF and Bare Sn at 1C. (e) Cycling performance of Sn@NaF and Bare Sn at 5C. (f) Comparison of cyclic performance between the Sn@NaF cell and other Sn cells. (g) Rate performance at 25°C of Sn@NaF and Bare Sn. (h) Comparison of rate performance between the Sn@NaF cell and other Sn cells.

The first three cyclic voltammetry (CV) curves of Bare Sn and Sn@NaF are displayed in Fig. S10. A cathodic peak emerges at ∼0.85 V in the first scan of both samples, which corresponds to the formation of the SEI film. Note that the peak intensity of Bare Sn is evidently higher than that of Sn@NaF, implying that much more SEI film is formed, which can account for the lower ICE of Bare Sn compared with Sn@NaF. Additionally, the peak intensity of Bare Sn gradually decreases in the following scans. In contrast, the CV curves of Sn@NaF almost overlap, indicating its high electrochemical reversibility and stability. The CV measurements are in good agreement with the discharge/charge profiles.

Long lifespan is an essential property of SIBs for large-scale energy storage applications, and the insufficient lifespan is also the main shortcoming of alloy anodes in comparison with HC anodes. Hence, the long-term cycling stability of Sn@NaF was investigated. Figure 2e shows that Sn@NaF delivers a specific capacity of 449 mAh g^–1^ after 4500 cycles, corresponding to a capacity retention of 81%. In contrast, Bare Sn only maintains a capacity retention of 63% over 900 cycles. The Sn@NaF anode in this work not only exhibits comparable cycling performance to the HC anode, but also has higher specific capacity and better high-rate performance, justifying its competitiveness in energy storage applications. To demonstrate the advantages, the overall electrochemical performances of the Sn@NaF anode and other reported bulk Sn-based anode materials are comparatively evaluated (Fig. 2f). It can be clearly observed that the Sn@NaF anode outperforms counterparts in terms of specific capacity, retention and cycle life [38,48–55].

The rate performance tests in Fig. 2g show that Sn@NaF exhibits remarkable rate capability, delivering capacities of 752, 740, 717, 680 and 594 mAh g^–1^ at 0.1C, 0.5C, 1C, 2C and 5C, respectively. When the current rate is reverted to 0.1C, the capacity is fully recovered. In contrast, Bare Sn manifests inferior rate performance, delivering capacities of 670, 646, 624, 587 and 437 mAh g^–1^ under the abovementioned current rates. Figure 2h further reveals that the rate performance of Sn@NaF anode is far superior to other bulk Sn-based anode materials reported so far. To the best of our knowledge, this is the first report of bulk Sn-based anode with such excellent performance in terms of long lifespan and high-rate capability. The significantly improved electrochemical performance can be attributed to the NaF coating, which not only alleviates the side reaction by isolating the active Sn particle from direct contact with the electrolyte, but also effectively buffers the volume change during the repeated sodiation/desodiation [38,48–55].

Subsequently, multi-sweep rate CV tests were performed on Sn@NaF to elucidate its charge storage mechanism (Fig. S11a). Sn@NaF exhibits well-preserved CV curves with tiny voltage polarization with the increase of sweep rate, indicative of excellent electrochemical reversibility. The charge storage dynamics was evaluated using the power-law relationship *i* = *a* × *v^b^*, where *i* and *v* denote the response current and scan rate, respectively, and *a* and *b* are constants. By transforming the equation to log(*i*) = *b* × log(*v*) + log(*a*) and calculating the slope, the *b* values for the labeled peaks I and II are determined to be 0.55 and 0.59, respectively (Fig. S11b), signifying that the charge storage mechanism of Sn@NaF is predominantly diffusion-controlled.

To further quantify the Na^+^ diffusion kinetics, Galvanostatic Intermittent Titration Technique measurements were conducted on Bare Sn and Sn@NaF, and the diffusion coefficient was calculated (Fig. S12). Throughout the entire potential range, the diffusion coefficient of Sn@NaF is consistently higher than that of Bare Sn. The electrochemical impedance spectroscopy (EIS) of Bare Sn and Sn@NaF electrode after five cycles (Fig. S13) shows that Sn@NaF has lower SEI resistance (*R*_SEI_) and charge transfer resistance (*R*_ct_), consequently demonstrating a superior rate performance. The improved reaction kinetics is associated with NaF coating, which prevents the breakage and pulverization of Sn particles during cycling, and eventually eliminates the repeated damage/regeneration of SEI.

Electrochemical tests show that the NaF coating significantly improves the performance of bulk Sn. This is because the presence of the coating can prevent the Sn from breaking due to excessive expansion stress, providing a large hoop stress and reducing the side reactions of the Sn. To intuitively observe the buffer effect of the NaF coating, *in situ* SEM test was performed on the Sn@NaF and Bare Sn electrode during discharge.

The test principle is shown in Fig. S14 [56]. As can be seen from Fig. 3, the volume of the Bare Sn single particle keeps increasing during the sodiation process, accompanied by the generation of microscopic lattice expansion, and then breaks and pulverizes accompanied by another macroscopic expansion (Fig. 3a–c). The Bare Sn after sodiation was sliced, and the cross section was characterized by SEM (Fig. 3d). It can be seen that the cracks run through the entire particle, so that more fresh surfaces are exposed, resulting in repeated growth of SEI and continuous consumption of electrolyte. Besides, the broken particles will lose electrical contact with the conductive carbon and current collector, leading to rapid capacity decay [57,58]. In contrast, the NaF layer of the Sn@NaF can alleviate and evenly distribute the expansion stress, resulting in stable lattice expansion. At the same time, the coating layer generates a huge hoop stress that prevents the Sn particles from breaking (Fig. 3e–g). Consequently, the Sn@NaF particle remains intact with tiny cracks (Fig. 3h), which benefits the structural integrity upon cycling.

**Figure 3.**
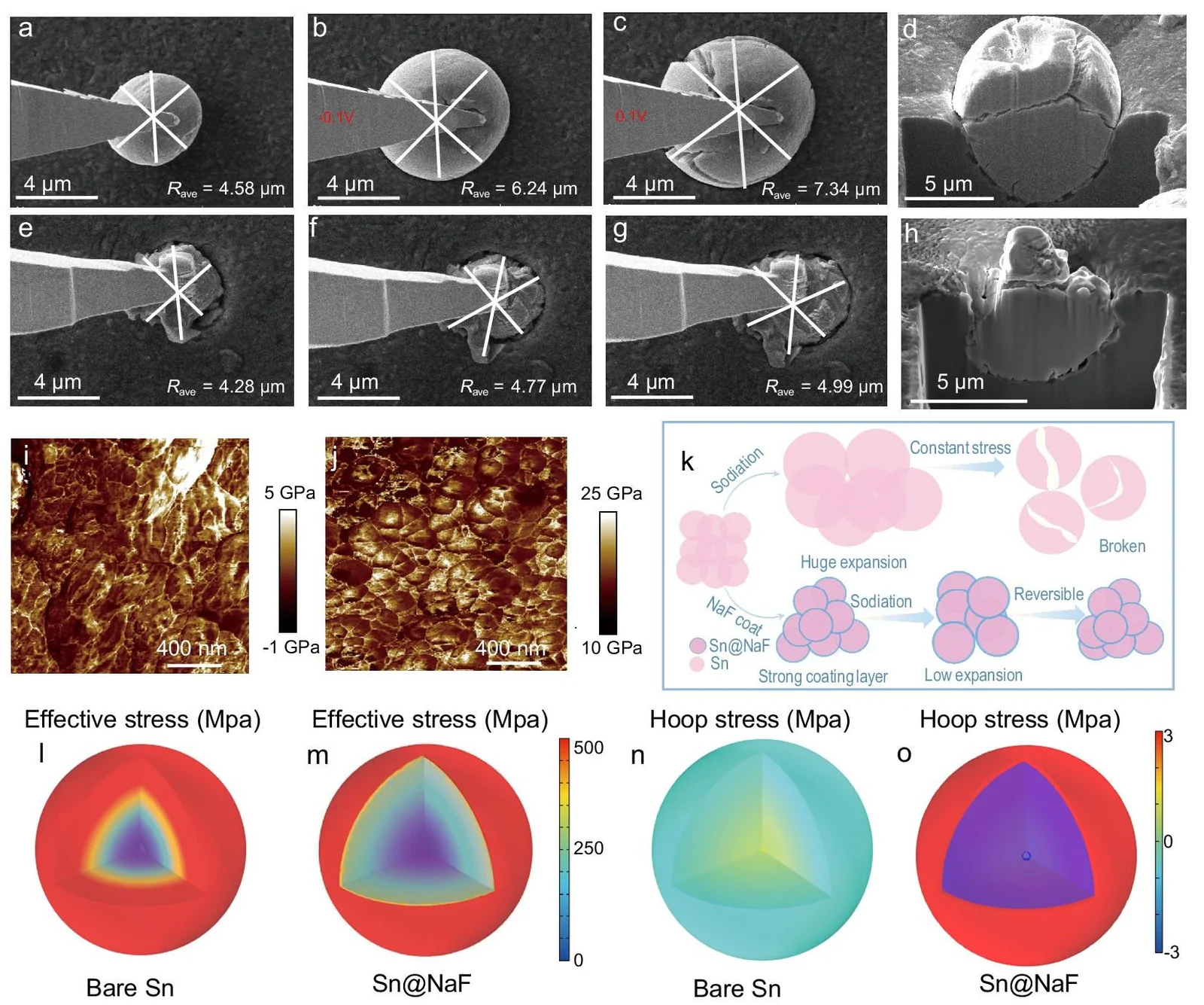
*In situ* studies and theoretical simulations of Sn@NaF and Bare Sn. (a–c) *In situ* SEM images for the sodiation of Bare Sn. (d) Cross-sectional SEM image of the Bare Sn. (e–g) *In situ* SEM images for the sodiation of Sn@NaF. (h) Cross-sectional SEM image of the Sn@NaF. (i and j) Young’s modulus of electrodes made of Bare Sn and Sn@NaF. (k) Schematic diagram of the cycle mechanism of Bare Sn and Sn@NaF. (l–o) Chemo-mechanical simulated effective stress (l and m) and hoop stress (n and o) in the cross sections of the fully sodiated Bare Sn and Sn@NaF particles.

Figure S15 compares the volume expansion rate and diameter change of Bare Sn and Sn@NaF at different discharge depths. It can be seen that both volume expansion rate and diameter of Bare Sn increase rapidly along sodiation, while the growth rate of Sn@NaF is significantly lower than that of Bare Sn. The morphological evolution of both anodes can be vividly observed by SEM tests at the first discharge, first charge and after 100 cycles (Fig. S16a–h). The Bare Sn particles experience severe cracking and pulverization; hence almost no complete spherical particles can be seen after 100 cycles. In sharp contrast, Sn@NaF can maintain the original morphology at different charge and discharge stages, further demonstrating the beneficial effect of NaF coating on the structural stability of the anode material.

From the above results, it can be proven that the volume expansion of Sn is composed of two parts: the crystal lattice volume expansion after the sodiation of Sn at the microscopic level, and the pore volume generated from the volume expansion of Sn exceeding its strength limit, causing it to break at the macroscopic level. Figure S17 shows the *in situ* XRD pattern of Sn. The appearance of the Na_15_Sn_4_ peak indicates the complete sodiation of Sn. The results of *in situ* SEM and *in situ* XRD indicate that during the crystal cell expansion process from Sn to Na_15_Sn_4_, the NaF coating layer can effectively alleviate and disperse the expansion stress. Moreover, the compressive stress of NaF causes that Sn@NaF only undergoes microscopic volume expansion during the sodiation process and does not produce macroscopic volume expansion. Atomic force microscopy was employed to characterize the mechanical properties of the Bare Sn and Sn@NaF. As illustrated in Fig. 3i and j, the Sn@NaF electrode sheet exhibits a significantly higher Young’s modulus (10.4 GPa) than the uncoated counterpart (1.5 GPa). This enhancement can be attributed to the inherently higher Young’s modulus of NaF relative to Sn, endowing the Sn@NaF electrode with better anti-deformation capability due to the alleviation of the expansion stress by the NaF layer [59,60]. Consequently, the NaF layer effectively mitigates the mechanical stress induced by the volume expansion/extraction during the sodiation/desodiation processes, thereby guaranteeing stable structure upon long-term cycling. Figure 3k presents a schematic illustration of the functional role of the cell wall structure. The NaF coating, with its high Young’s modulus, forms a robust barrier layer on the surface of Sn particles. This layer uniformly distributes the volume expansion stress during Sn sodiation, acting as a resistive layer against the outward expansion of Sn. Analogous to a balloon, the NaF coating combines restrictive and elastic properties, effectively constraining the volume increase upon sodium intercalation. As a result, the likelihood of bulk Sn particle fracture and pulverization is significantly reduced, thereby substantially enhancing the cycling stability of the anode material.

To elucidate the underlying mechanism for the improved mechanical stability of the Sn@NaF composite anode material, we systematically investigated the dimensional evolution, Na^+^ concentration distribution and stress state evolution of a single particle during sodium insertion via finite element modeling. A three-dimensional single-particle model was constructed, wherein Na^+^ diffusion was governed by Fick’s second law, and the stress–strain response was described using a Neo-Hookean hyperelastic constitutive model. We calculated the stress distribution when Bare Sn and Sn@NaF were both sodiated according to the theoretical volume expansion (420%). Figure 3l–o presents the effective stress distributions of fully sodiated Sn@NaF particles and Bare Sn particles. Iso-surfaces of Na^+^ concentration indicate that during the initial stage of sodiation, Na^+^ are preferentially adsorbed on the particle surface at a high diffusion rate, and subsequently migrate inward along the radial direction (Fig. S18a and b). In the Bare Sn particles, the volumetric expansion is largely unconstrained owing to the low Young’s modulus of the surrounding binder material. This results in significant outward expansion of the outer Sn region after sodiation, while the inner unsodiated Sn region is subjected to tensile forces induced by the expansion of the outer layer, leading to high effective stresses and tensile hoop stresses (Fig. 3l and n), ultimately causing plastic deformation and particle cracking. In contrast, the coating layer on the Sn@NaF particles possesses a high Young’s modulus, which imposes significant elastic constraint on the volumetric expansion of the outer Sn region upon sodiation. This constraint alters the expansion behavior from the predominantly outward expansion observed in pure Sn to a confined expansion in both outward and inward directions. As a result, the tensile effect on the inner unsodiated region is markedly alleviated, and the stress state shifts from being tensile-dominated to compressive-dominated (Fig. 3m and o). This confined expansion mode not only shortens the diffusion path of Na^+^ but also promotes a more uniform stress distribution within the particle, localizing stress concentration near the coating interface, thereby effectively suppressing crack initiation and propagation. Furthermore, during the early stage of sodiation, the Bare Sn particles exhibit a steep gradient in effective stress due to the significant differential expansion between the surface (high sodium concentration region) and the core (unsodiated region) (Fig. S18c). The NaF coating layer on the particles effectively reduces this stress gradient by constraining radial expansion, thereby mitigating stress-induced brittle fracture.

In summary, the NaF coating layer acting as an artificial SEI layer enhances the mechanical stability of the Sn@NaF anode during electrochemical cycling by reducing stress heterogeneity, suppressing tensile stresses and alleviating stress gradients. The NaF coating enables the Sn particles to undergo the lattice expansion from Sn to the Na_15_Sn_4_ alloy. Benefiting from the high hoop stress of NaF, the Sn particles are prevented from breaking and pulverizing, thus avoiding the generation of macroscopic volume expansion. Consequently, Sn@NaF only experiences uniform microscopic lattice volume expansion, eventually manifesting a very high CCE and cyclic stability.

To elucidate the effect of NaF coating on the formation of SEI film, X-ray photoelectron spectroscopy (XPS) was performed on cycled Bare Sn and Sn@NaF electrodes (Fig. 4a–c). The results reveal that Sn@NaF forms a thin and uniform SEI film after the first cycle (Fig. 4d), and the subsequent SEI growth is suppressed by the NaF coating layer. The physical barrier provided by NaF prevents direct contact between Sn and the electrolyte, minimizing side reactions and promoting the formation of a robust SEI layer. In contrast, the SEI film on Bare Sn contains a significantly higher proportion of organic sodium salts. The coating layer of NaF is beneficial for the formation of a thin and dense SEI film, thereby enhancing the stability of the material. This discrepancy stems from the distinct SEI formation behavior. Bare Sn undergoes repeated fragmentation during cycling, leading to continuous SEI regrowth on newly exposed surfaces. This results in a thick and uneven SEI film (Fig. 4f) dominated by organic sodium salts, which impedes the Na^+^ diffusion and exacerbates capacity decay. As corroborated by former studies, continuous anode fragmentation and SEI regrowth are detrimental to the cycling performance, whereas an NaF-rich SEI film exhibits higher mechanical strength and ion-conduction efficiency [57,58]. Schematic illustrations in Fig. 4e and g succinctly depict the contrasting SEI evolution and structural stability mechanisms between the two electrodes, underscoring the critical role of the NaF coating in suppressing the incrassation of SEI and enhancing long-term cycling performance.

**Figure 4.**
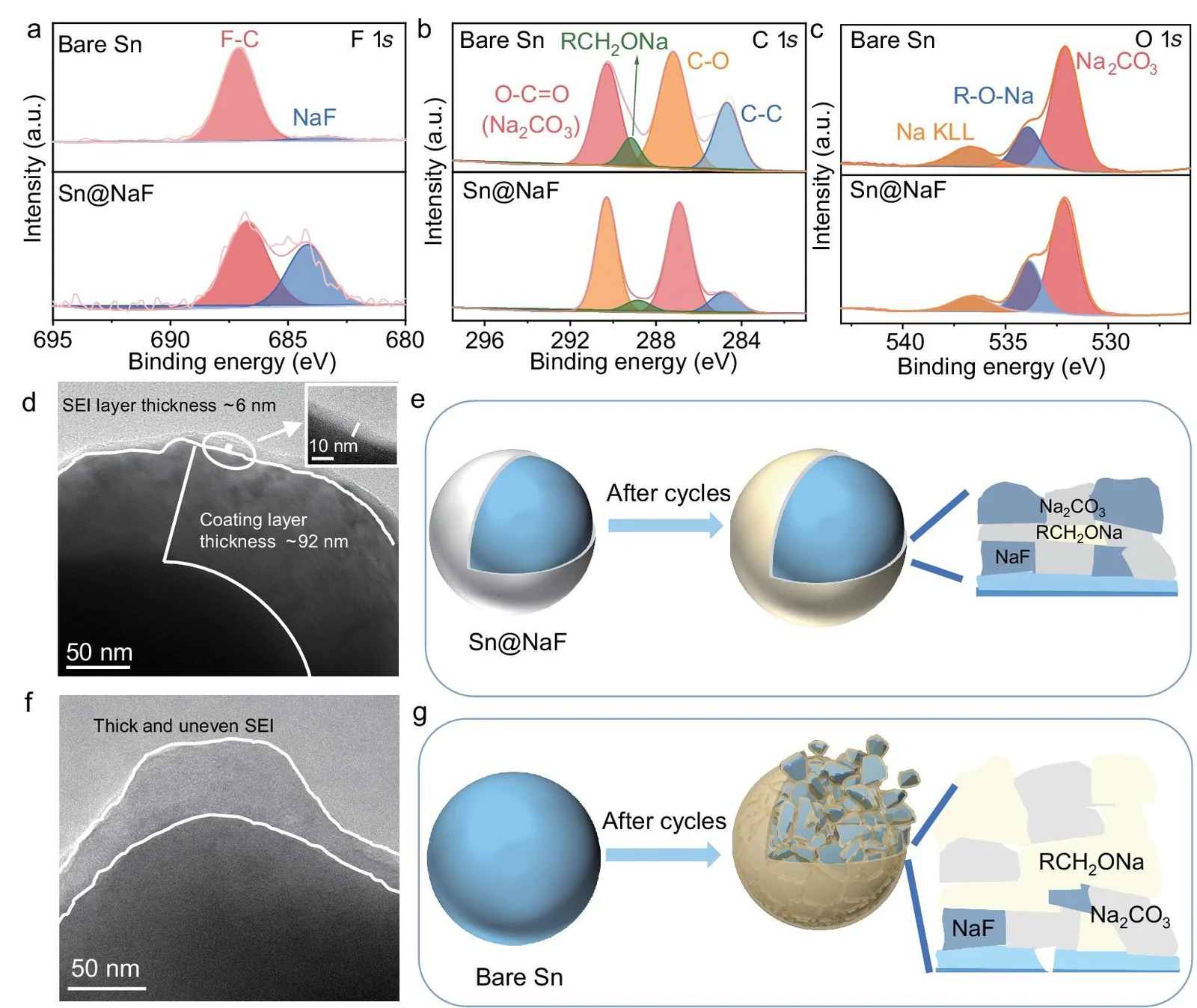
Characterizations of the electrode/electrolyte interface. (a–c) High-resolution F 1*s*, C 1*s* and O 1*s* XPS spectra of cycled Bare Sn and Sn@NaF. (d and f) TEM images of cycled Sn@NaF (d) and Bare Sn (f). (e and g) Schematic illustration of the SEI evolution after cycling.

To evaluate the practical applicability of Sn@NaF, coin full cells were assembled using Sn@NaF as the anode, self-synthesized NVP as the cathode and NaPF_6_ dissolved in diethylene glycol dimethyl ether as the electrolyte. The XRD pattern and cycling performance of the synthesized NVP are presented in Fig. S19a and b, confirming its crystalline structure and electrochemical stability. Prior to full-cell assembly, both electrodes were pre-activated for two charge–discharge cycles in half-cell configurations.

The initial charge–discharge curves of the Sn@NaF||NVP full cell are shown in Fig. 5a. The full cell delivers a discharge capacity of 696 mAh g^–1^ (corresponding to the 94.4% of utilization of Sn@NaF anode) with an average operating voltage of 3.1 V. Multiple distinct charge–discharge voltage plateaus are observed, suggesting the reversible sodiation/desodiation process in both the NVP cathode and the Sn@NaF anode. Rate performance test was performed at current densities ranging from 0.1C to 5C, and the corresponding power and energy densities were calculated (Fig. 5b). The full cell delivers specific capacities of 708, 665, 634, 586 and 559 mAh g^–1^ based on the Sn@NaF anode, respectively. Notably, the full cell achieves an energy density of up to 262 Wh kg^–1^ at 0.2C (based on the total active mass of anode and cathode). Even at a high current density of 5C, it manifests an energy density of 192 Wh kg^–1^ with a high-power density of 2608 W kg^–1^, outperforming previously reported full cells in both metrics (Fig. 5c) [61–64].

**Figure 5.**
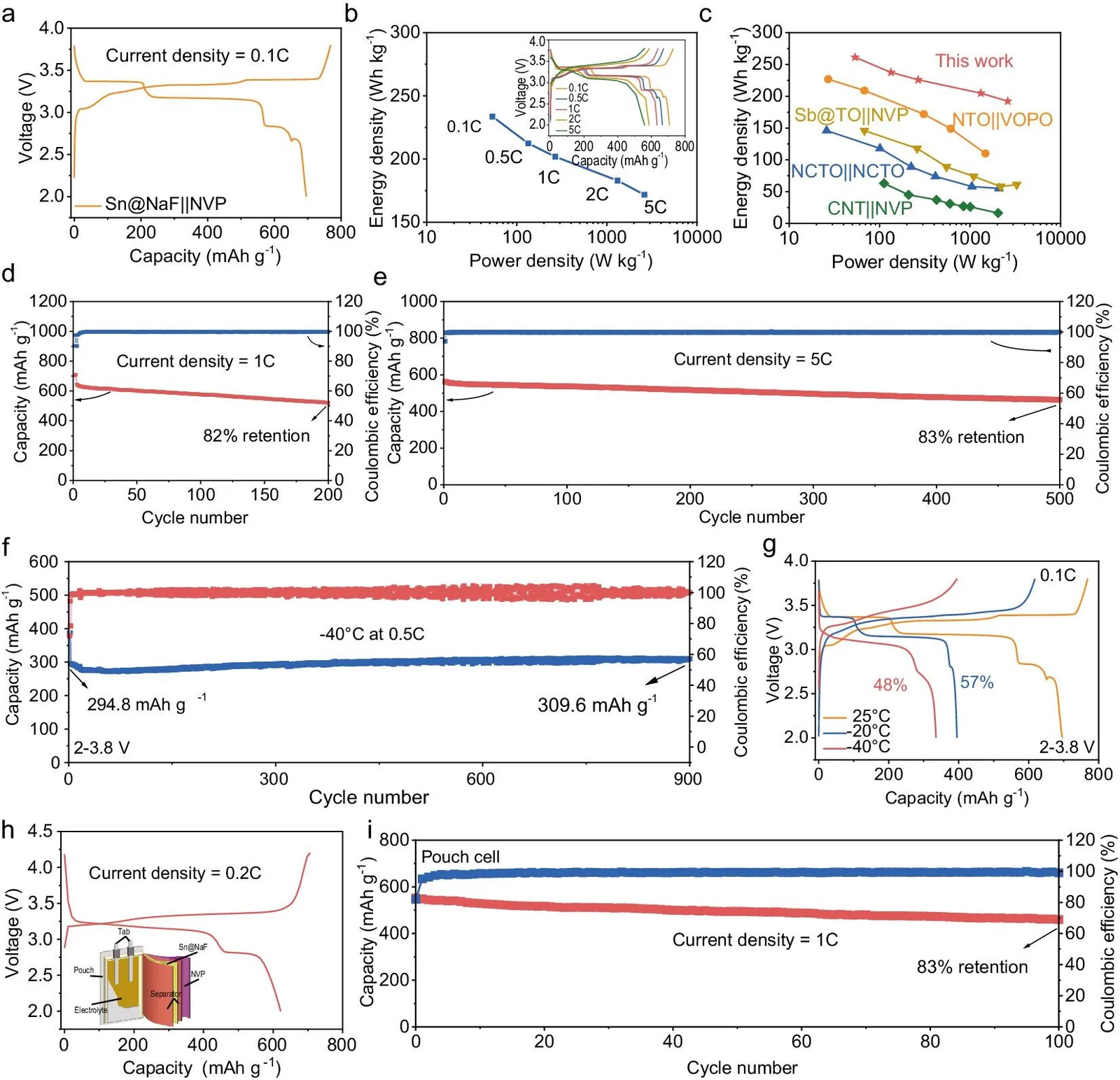
Electrochemical performance of the Sn@NaF||NVP full cell, 1C = 847 mA g^–1^. (a) Galvanostatic discharge/charge profiles of the Sn@NaF||NVP full cell at 0.1C. (b) Rate capability, energy density and power density of the Sn@NaF||NVP full cell. (c) Ragone plots of the Sn@NaF||NVP full cell and other sodium-ion full cells [57–60]. (d and e) Cycling performance of the Sn@NaF||NVP full cell at 1C (d) and 5C (e). (f) Cycling performance of the Sn@NaF||NVP full cell at 0.5C at −40°C. (g) Galvanostatic discharge/charge profiles of the Sn@NaF||NVP full cell at 25, −20 and −40°C at 0.1C. (h) Initial charge/discharge voltage curve of the Sn@NaF||NVP pouch cell at 0.2C. (i) Cycling performance of the Sn@NaF||NVP pouch cell at 1C.

The cycling performance tests in Fig. 5d show that the full cell exhibits capacity retention of 82% after 200 cycles at 1C. Even at 5C, 83% of its initial capacity can be reserved after 500 cycles (Fig. 5e), indicating an outstanding cycling stability. The full cell also demonstrates exceptional low-temperature performance. Note that the full cell sustains stable cycling for 900 cycles at −40°C (Fig. 5f). The charge–discharge curves show slight voltage shift at −20°C and −40°C (Fig. 5g), and the discharge capacities at −20°C and −40°C are 395 and 335 mAh g^–1^, respectively, corresponding to 57% and 48% of the capacity at room temperature. At the onset of cycling, immediate exposure to a low-temperature environment induces pronounced polarization in the battery, with an initial capacity of 294.8 mAh g^–1^ followed by a gradual decrease. As cycling proceeds, this polarization diminishes, and the capacity exhibits a progressive recovery, peaking at 309.6 mAh g^–1^ by the 900th cycle. These findings demonstrate its promising potential for applications in extremely cold environments.

Given the outstanding performance of Sn@NaF in the full cell, Sn@NaF||NVP pouch cells were further assembled to assess the practical applicability of the Sn@NaF anode. Figure 5h shows the charge–discharge curve of the initial cycle of the pouch cell (inset is the diagram of the pouch cell) between 2.0 to 4.2 V. It can be seen that the first discharge capacity of the cell is 622 mAh g^–1^ (calculated based on the weight of the anode), with an ICE of 87% and two discharge voltage platforms at 3.2 and 2.8 V, respectively. Also, it offers a stable cycling performance at 1C with a capacity retention of 83% after 100 cycles (Fig. 5i), signifying the promising application prospect of the Sn@NaF anode.

## CONCLUSIONS

In this study, an NaF coating layer was successfully grown *in situ* on bulk Sn via the solution supersaturation mechanism, endowing the Sn anode with remarkable structural and cycling stability during the sodiation/desodiation processes. The high Young’s modulus of the NaF coating brings a strong mechanical confinement effect, significantly mitigating the mechanical stress generated during Sn sodiation. By effectively reducing the volume expansion rate of Sn, it preserves the structural integrity of the anode material throughout repeated cycles, thereby enabling the outstanding long-term cycling stability of Sn@NaF. Electrochemical tests demonstrate that the Sn@NaF anode delivers a high reversible capacity of 739.9 mAh g^−1^ at 0.1C, and retains 81% of its initial capacity after 4500 cycles at 5C. The NaF coating also participates in the formation of a thin, uniform and dense SEI film, which effectively suppresses side reactions. Hence, a high ICE of 84% is achieved. More significantly, the Sn@NaF anode also demonstrates excellent electrochemical performance in a full cell when coupling with the NVP cathode. The Sn@NaF||NVP coin full cell retains a specific capacity of 464 mAh g⁻¹ after 500 cycles at 5C, corresponding to a capacity retention of 83%. Note that the Sn@NaF-based full cell exhibits outstanding low-temperature stability, sustaining 900 stable cycles at −40°C. Particularly, the assembled Sn@NaF||NVP pouch cell shows an ICE of 87% with a stable life in 100 cycles. These findings highlight that the mechanical confinement effect induced by high Young’s modulus NaF coating significantly enhances the electrochemical performance of bulk Sn anodes, thereby offering a promising applicable high-capacity anode for high-energy-density SIBs.

## Supplementary Material

nwag428_Supplemental_File

## Data Availability

The detailed experimental reagents and methods can be found in the Supplementary data.
