# Supplementary material for: High Young’s modulus NaF coating on bulk Sn: mechanical confinement effect drives durable sodium storage by alleviating (de)sodiation stress
Source: Natl Sci Rev. 2026 Jul 11;13(19):nwag428. doi: 10.1093/nsr/nwag428 (PMC13715433; doi:10.1093/nsr/nwag428)
Supplement: Supplementary file 1 [file nwag428_supplemental_file.pdf]

# Supporting information

## High Young's modulus NaF coating on bulk-Sn: mechanical confinement effect drives durable sodium storage by alleviating (De) sodiation stress

Huiqian Xiong[1], Benchi Li[1], Jianing Wang[1], Yanxia Wang[1], Zhaoyu Rong[2], Yakun Tang[3], Yue Zhang[3], Yongjin Fang[1], Zhongxue Chen[4,*], Jianyu Huang[2,*] and Yuliang Cao[1,3,5,*]

[1]College of Chemistry and Molecular Sciences, Hubei Key Laboratory of Electrochemical Power Sources, Wuhan University, Wuhan 430072, China;
[2]Clean Nano Energy Center, State Key Laboratory of Metastable Materials Science and Technology, Yanshan University, Qinhuangdao 066004, China;
[3]State Key Laboratory of Chemistry and Utilization of Carbon Based Energy Resources; College of Chemistry, Xinjiang University, Urumqi 830017, China;
[4]School of Power and Mechanical Engineering, Ministry of Education, Wuhan University, Wuhan 430072, China and [5]Perception and Effectiveness Assessment for Carbon-neutrality Efforts, Engineering Research Center of Ministry of Education, Institute for Carbon Neutrality, Wuhan University, Wuhan 430072, China

***Corresponding authors.** E-mails: ylcao@whu.edu.cn; zxchen_pmc@whu.edu.cn; jhuang@ysu.edu.cn

## Methods

**Preparation of Sn@NaF**: Sn powder (≥99%, 100 mesh, metals basis; Energy Chemical, China) was ultrasonically washed in ethanol to ensure the surface was clean and then dried as a pretreatment step. Then, Sn powder is dispersed in a uniform aqueous solution of NaF (3%NaF by the mass of Sn powder) to form a slurry. Subsequently, it was slowly dried at 40°C in an air-drying oven, when the NaF concentration reached a supersaturated state, triggering the growth of the coating layer with bulk Sn crystals acting as nucleation cores.
**Preparation of $Na_3V_2(PO_4)_3$**: In a beaker, 0.8mmol of citric acid, 0.6mmol of sodium dihydrogen phosphate ($NaH_2PO_4$), and 0.4mmol of ammonium vanadate ($NH_4VO_3$) were added. The mixture was heated to 60°C in an aqueous solution and constantly stirred until evenly mixed. The resulting mixed solution was then spray-dried, with the outlet temperature set at 90 degrees. The obtained powder was calcined at 700°C in an argon atmosphere for 10 hours.

## Electrochemical Performance

Bare Sn and Sn@NaF were coated respectively. The material, sodium carboxymethyl

cellulose CMC (2.5 wt %, $H_2O$), and SuperP were mixed in a 7:1:2 ratio to form a uniform slurry, which was then coated onto copper foil and dried in an oven overnight. NVP, polyvinylidene difluoride PVDF (4wt%, NMP) and Acetylene Black were mixed in a weight ratio of 8:1:1 to form a uniform slurry which was then coated onto aluminum foil and dried overnight in an oven at 60℃. The obtained electrode sheets were respectively cut into circular sheets with a diameter of 12 cm and square sheets of 4 x 4 cm, which were used as the positive electrode sheets for the full-cell and the pouch-cell, respectively. The electrode sheets obtained from Sn@NaF were also cut to the same size as the negative electrode sheets. The amount of electrolyte added for each coin cell is 70μL. Then they were assembled into batteries, and electrochemical tests were conducted on them using $NaPF_6$ dissolved in ethylene glycol dimethyl ether (G2) as the electrolyte. The separator used is glass fiber.

## Material characterization

The specific surface area was calculated based on the $N_2$ adsorption isotherm by using the Brunauer-Emmett-Teller (BET)method (iPore 400). Scanning electron microscopy (SEM, Zeiss Merlin Compact, Germany) and transmission electron microscopy (TEM, FEI Tecnai G2 F20, America) were employed to observe the morphology and microstructure of the samples. And the element distribution uniformity was determined by EDS mapping analysis attached to the SEM. High-angle annular dark-field scanning transmission electron microscopy (HAADF-STEM) was performed using a JEM-F200. The crystallographic structure and diffraction peak positions of the samples were probed by an X-ray diffractometer (XRD, Bruker D8 ADVANCE) with a monochromatic Cu Ka X-ray source. The valence data of the sample were determined by X-ray photoelectron spectroscopy (XPS, Thermo Scientific ESCALAB Xi+, America). TOF-SIMS analyses were performed on a TESCAN Amber equipped with TOF-SIMS. To obtain a depth profile, an area (2 μm × 2 μm) was sputter etched using an Ga+ bean (30.0 kV, 50 pA). The numerical calculations in this paper have been done on the supercomputing system in the Supercomputing Center of Wuhan University. The in-situ SEM experiment was conducted in an FIB-SEM system (Helios G4 CX, Thermo Fisher Scientific). Atomic force microscopy (AFM) images were obtained using a PARK SYSTEMS NX10.

## Electrochemical measurements

Bare Sn refers to the commercial Sn particles, without prior treatments. The coin cells (CR2032) were assembled in an argon-filled glovebox (both water and oxygen content were less than 0.01 ppm) with glass fiber filters (Whatman GF/F) as separators and $NaPF_6$ dissolved in ethylene glycol dimethyl ether (G2) as electrolyte for electrochemical testing. Galvanostatic charging-discharging tests were conducted on the NEWWARE (CT-4008Tn-5V10mA-164) in a potential range between 0.01 and 1.0 V and cyclic voltammetry (CV) was performed on the CHI 760D electrochemical workstation. Galvanostatic charge/discharge measurements and galvanostatic intermittent titration technique (GITT) tests were performed on a LAND cycler (Wuhan Kingnuo Electronic Co., China). The electrochemical impedance spectroscopy (EIS)

data were collected in the AutoLab PGSTAT 128N (Eco Chemie, Netherlands) in the frequency range 0.1 to $10^5$ Hz with a perturbation amplitude of 5 mV.

**Chemo-mechanical Simulation Method**

A three-dimensional chemo-transport-mechanics coupled framework was implemented in MATLAB to investigate coating effects on sodiation-induced deformation. Two independent models were established: (i) a pure Sn particle model and (ii) a Sn@NaF core-shell configuration with NaF coating, both employing concentric spherical geometry. The Sn core possessed an initial radius $R_{core}=2.0\times10^{-6}$ m, while the NaF coating exhibited a thickness $t_{coat}=1.0\times10^{-7}$ m exclusively in the core-shell model. Sodium ion diffusion was governed by Fick's second law:

$$\frac{\partial c}{\partial t}=\nabla\cdot(D\nabla c)$$

where $c$ is the normalized $Na^+$ concentration, $t$ is time, and $D$ is the diffusion coefficient. A uniform diffusion coefficient $D = 1.0 \times 10^{-13}$ $m^2\cdot s^{-1}$ was applied throughout all domains. Initial conditions prescribed $c = 0$ within the particles, with Dirichlet boundary conditions (c=1) imposed at external surfaces and concentration/flux continuity maintained at core-coating interfaces.

The total strain tensor follows the decomposition:

$$\varepsilon_{ij}=\varepsilon_{ij}^{mech}+\varepsilon_{ij}^{chem}$$

where $\varepsilon_{ij}^{mech}$ denotes mechanical strain and $\varepsilon_{ij}^{chem}$ represents chemical strain. Chemical expansion was modeled isotropically:

$$\varepsilon_{ij}^{chem}=\beta(c-c_{ref})\delta_{ij}$$

where $\beta$ is the chemical expansion coefficient, $c_{ref}$ is the reference concentration, and $\delta_{ij}$ is the Kronecker delta. Mechanical equilibrium satisfies:

$$\nabla \cdot \sigma = 0$$

where $\sigma$ is the Cauchy stress tensor. Material behavior was described by a compressible Neo-Hookean hyperelastic constitutive law with Simo-Pister volumetric coupling, having the strain energy density function:

$$W(F)=\frac{\mu}{2}(I_1-3)-\mu\ln J+\frac{K}{2}(\ln J)^2$$

where $F$ is the deformation gradient, $J=\det F$, $I_1=\mathrm{tr}(F^{TF})$, $\mu$ is the shear modulus, and $K$ is the bulk modulus.

The Sn core material exhibited a Young's modulus of 25 GPa, Poisson's ratio of 0.30, and density of 7280 $kg\cdot m^{-3}$. The NaF coating possessed a Young's modulus of 75 GPa, Poisson's ratio of 0.30, and density of 2250 $kg\cdot m^{-3}$. The conductive binder, when explicitly included, demonstrated a Young's modulus of 1 GPa, Poisson's ratio of 0.30, and density of 1500 $kg\cdot m^{-3}$. The chemical expansion coefficient $\beta$ was specified as 0.6.

Simulation shows that the coating effectively reduces von Mises equivalent stress and circumferential tensile stress, thereby effectively suppressing particle fracture.

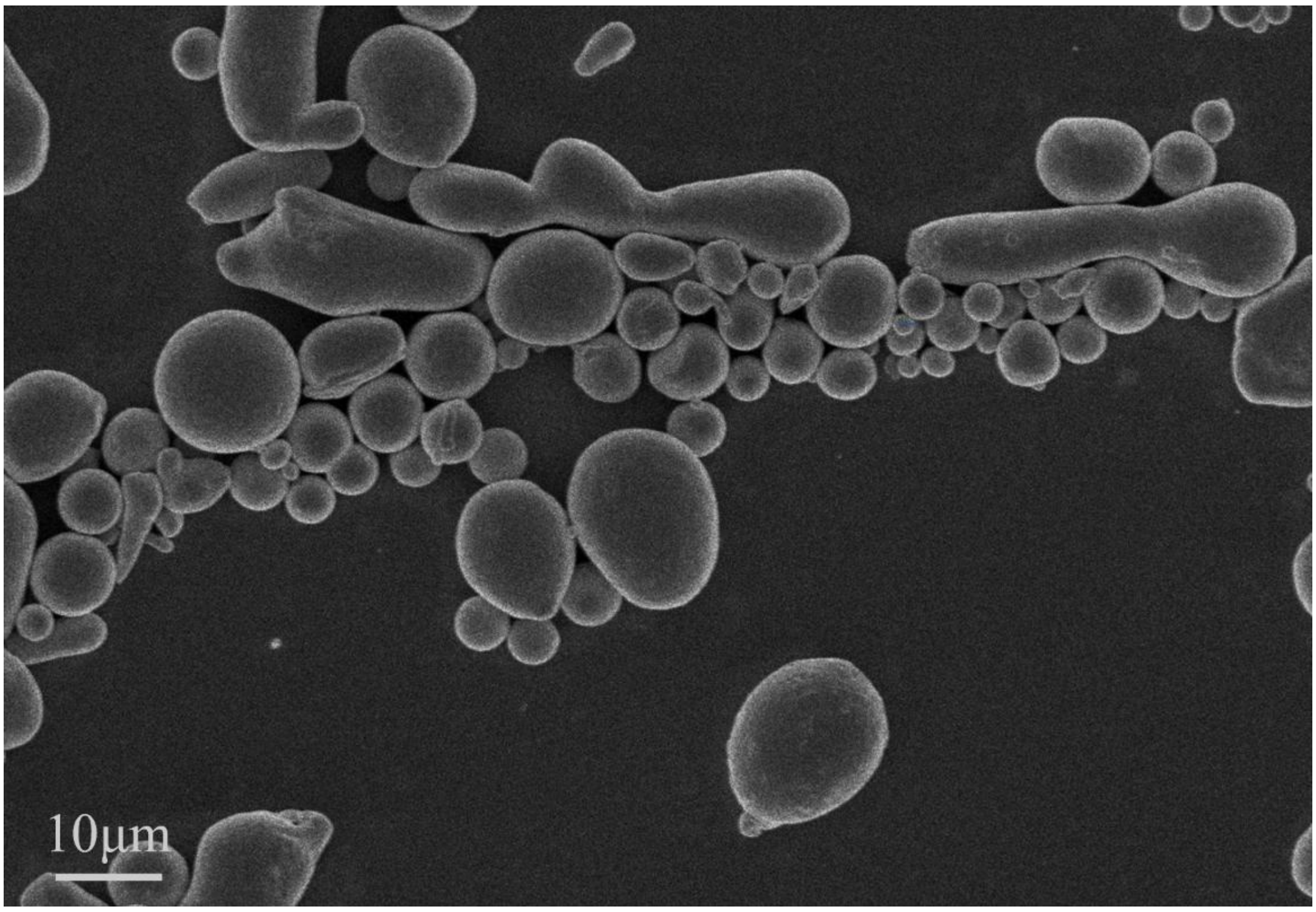


**Figure S1**. SEM images of Bare Sn.

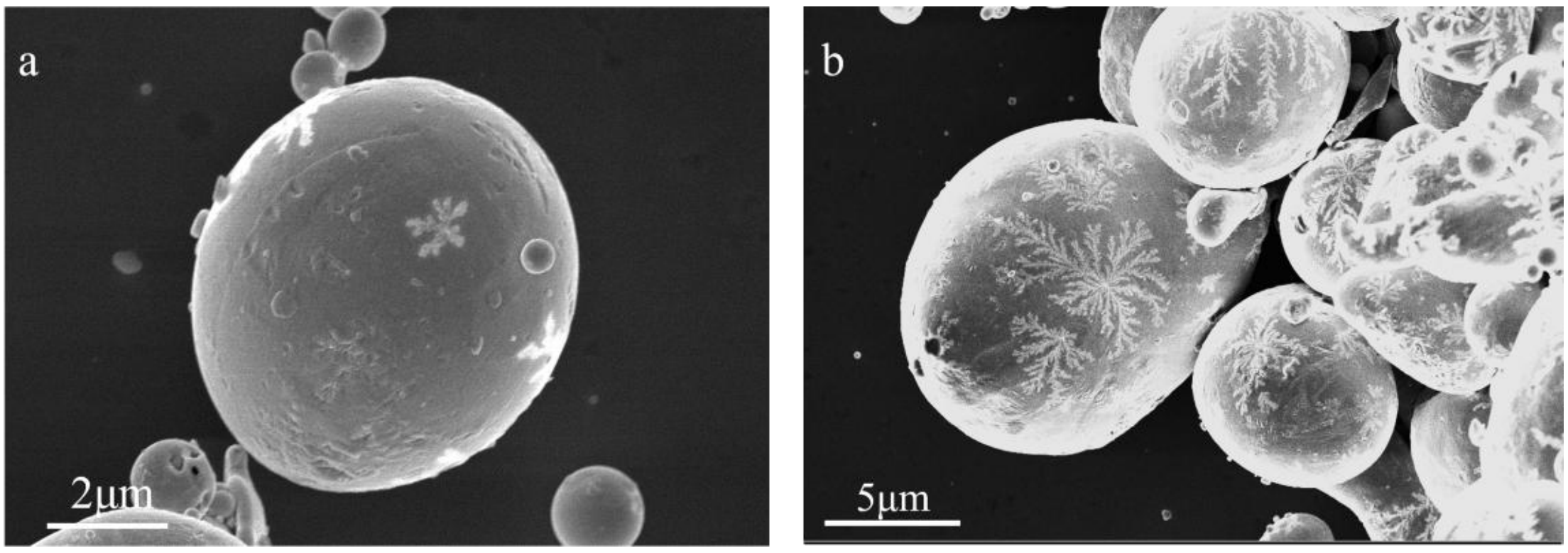


**Figure S2**. SEM images of the process of sodium fluoride coating Sn. (a) Crystallization begins at the defect. (b): NaF starts to grow and coat.

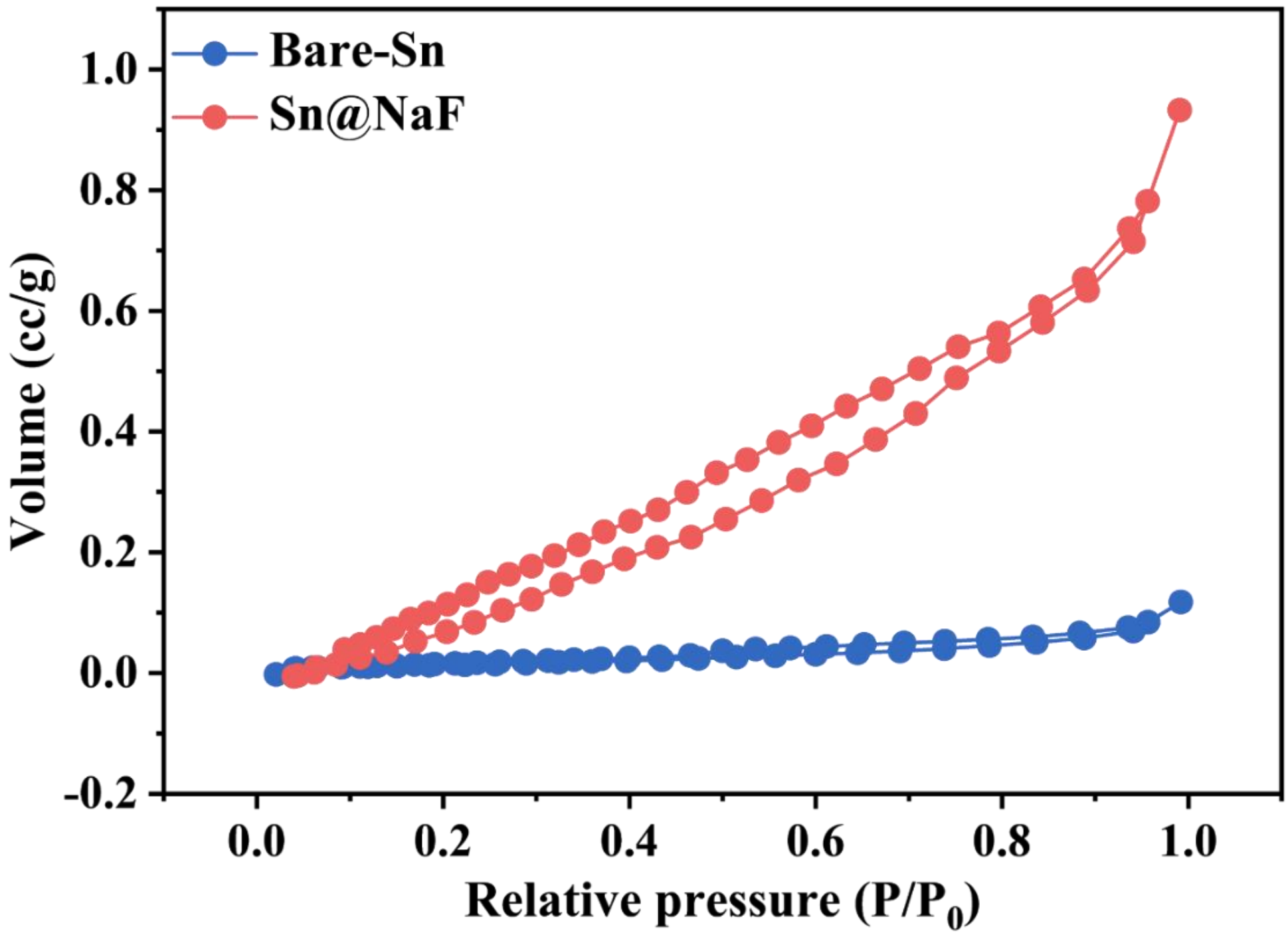


**Figure S3**. BET diagram of Bare Sn and Sn@NaF.

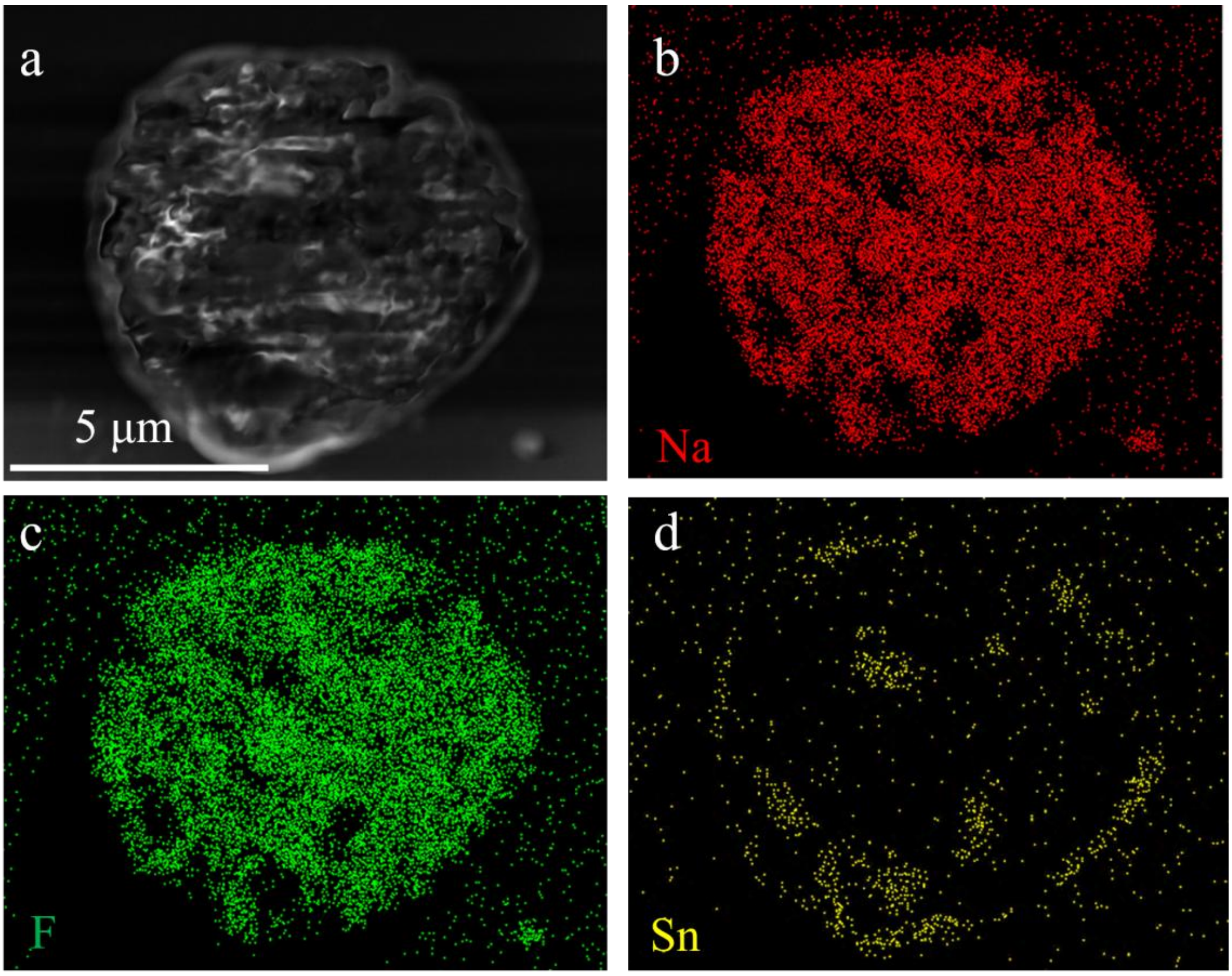


**Figure S4**. (a) SEM image and (b-d) corresponding element mapping of Sn@NaF.

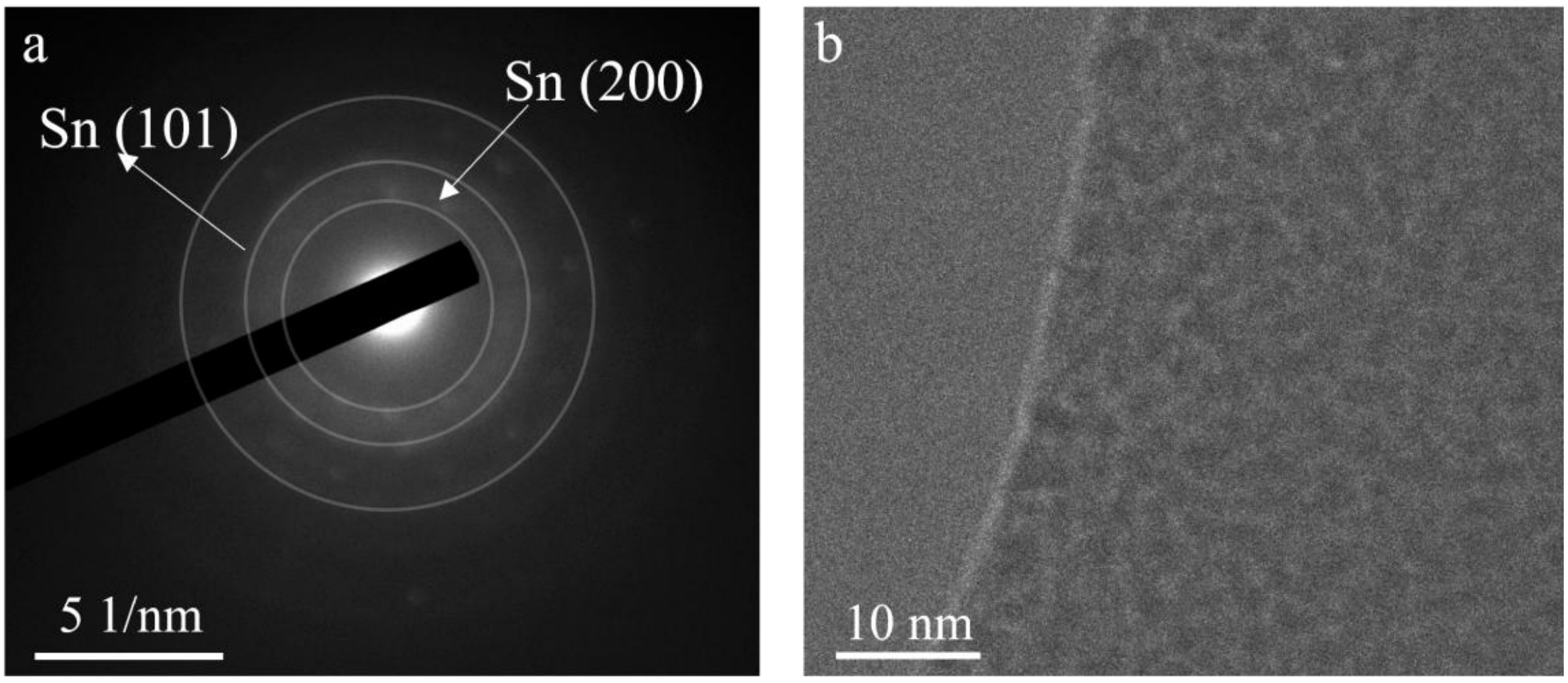


**Figure S5**. (a) SAED figure of Sn@NaF and (b) Lattice fringes of the coating layer of Sn@NaF.

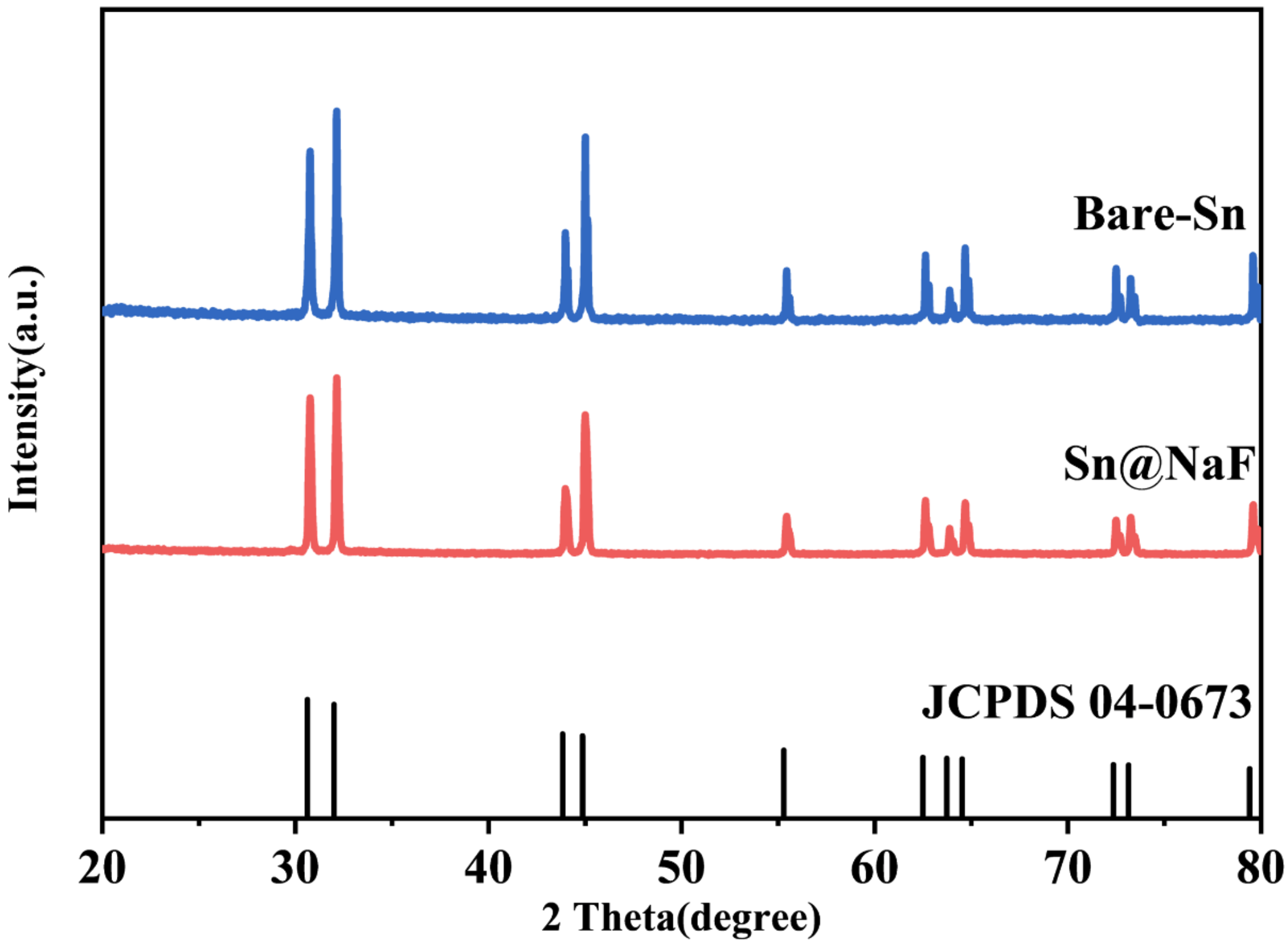


**Figure S6**. XRD patterns of Bare Sn and Sn@NaF.

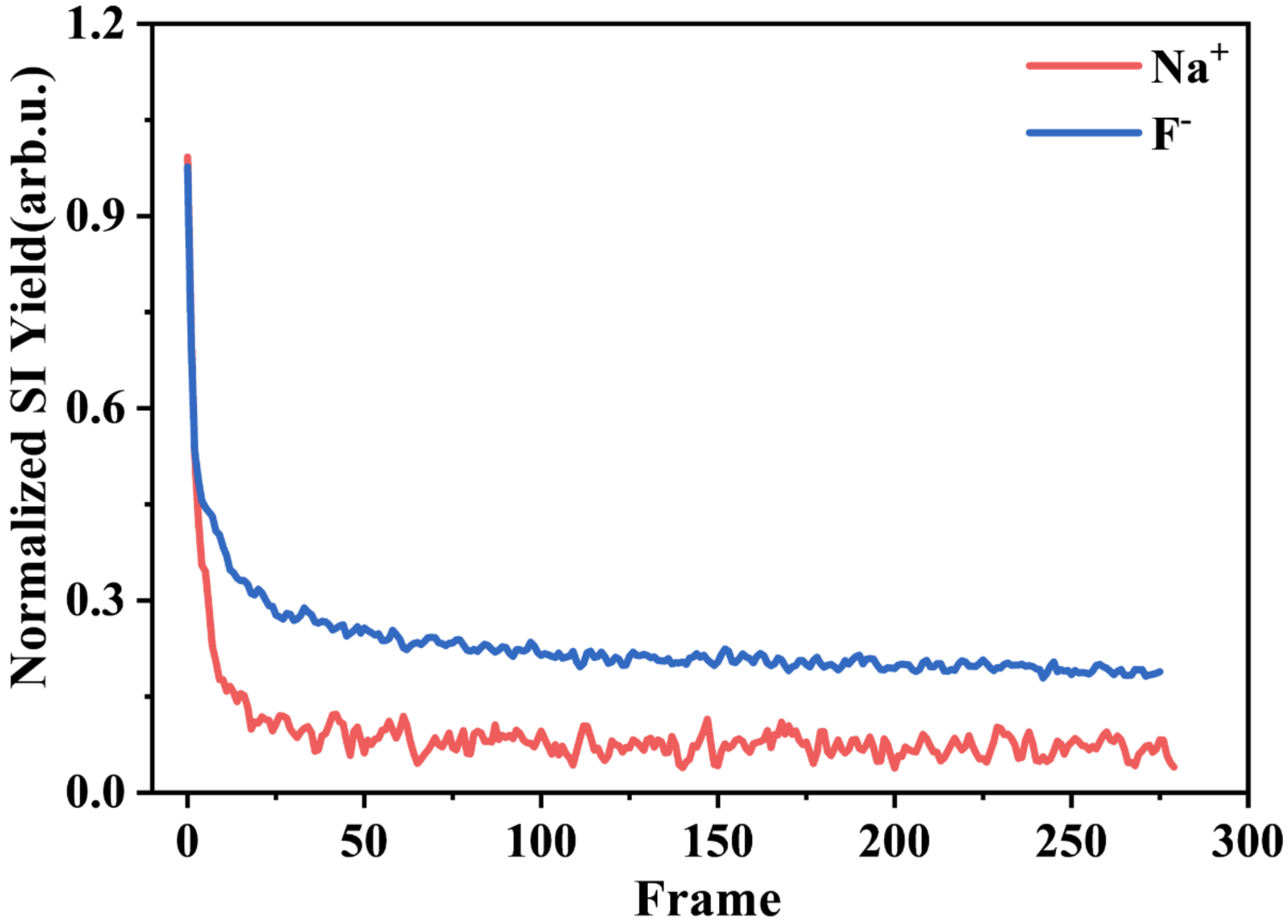


**Figure S7**. The depth distribution curves of $F^-$ and $Na^+$ with sputtering time.

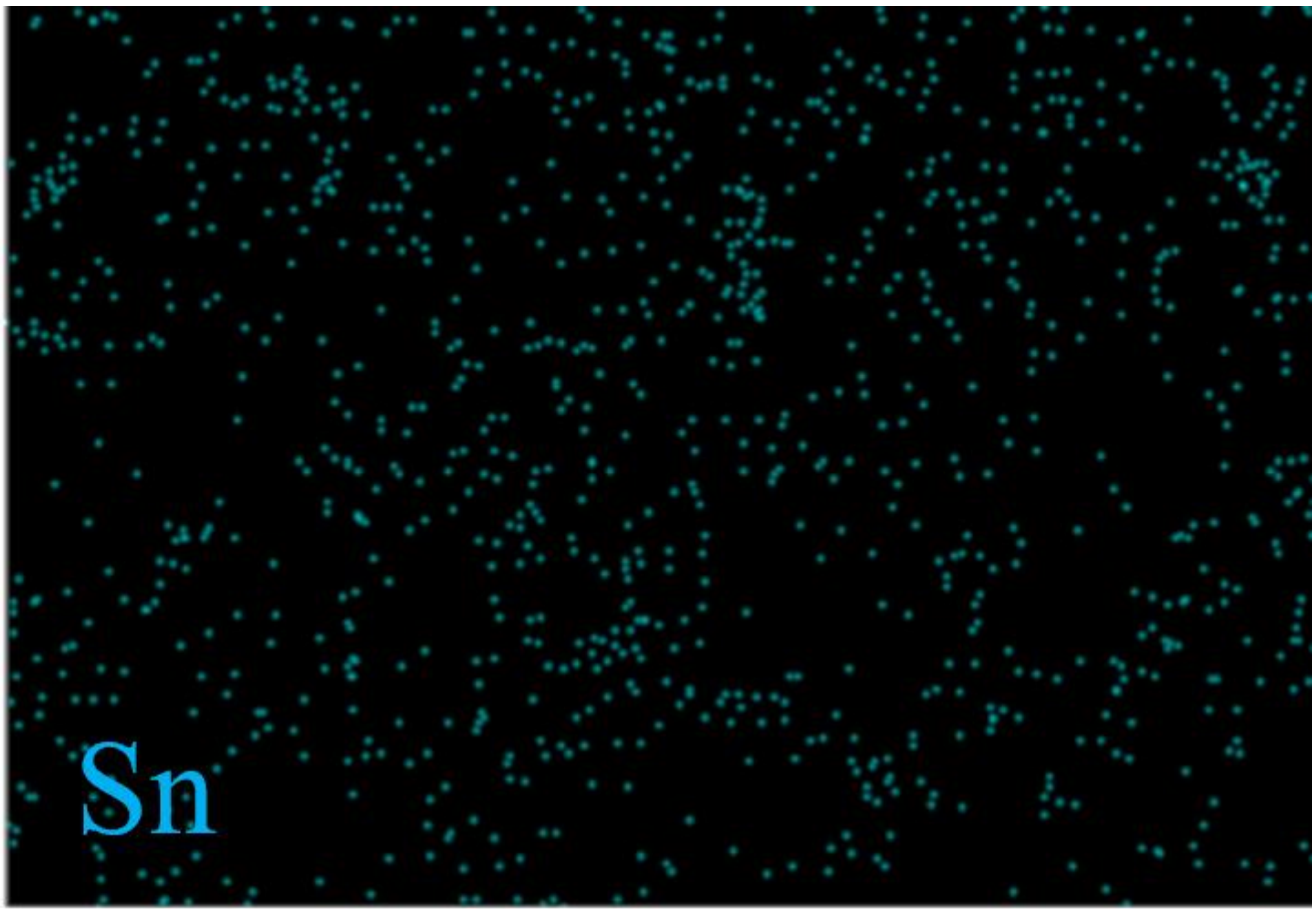


**Figure S8**. Sn elemental mapping of the electrode made of Sn@NaF.

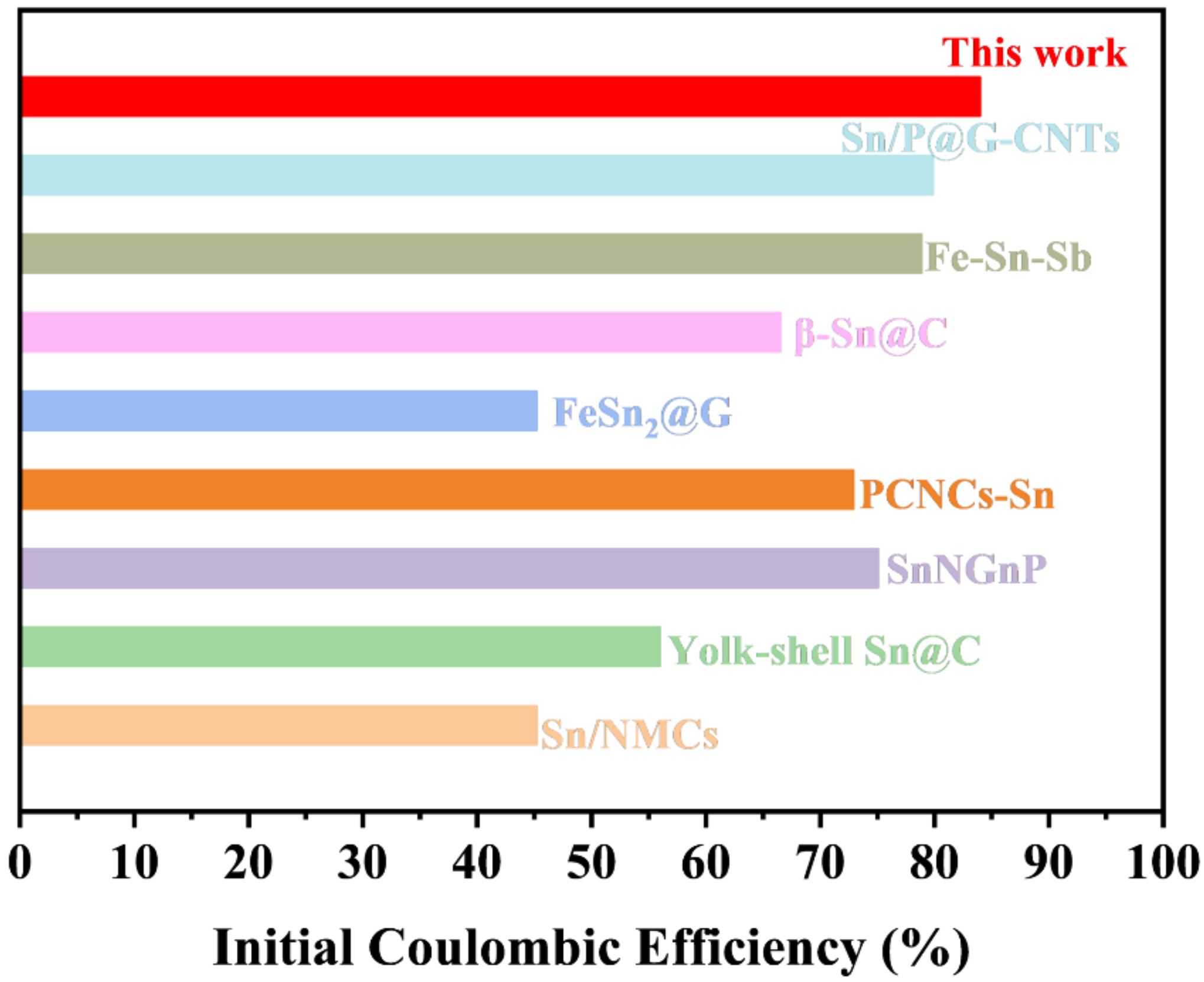


**Figure S9.** Comparison of ICE between Sn@NaF anode and other previously reported Sn-based anodes. [1–8]

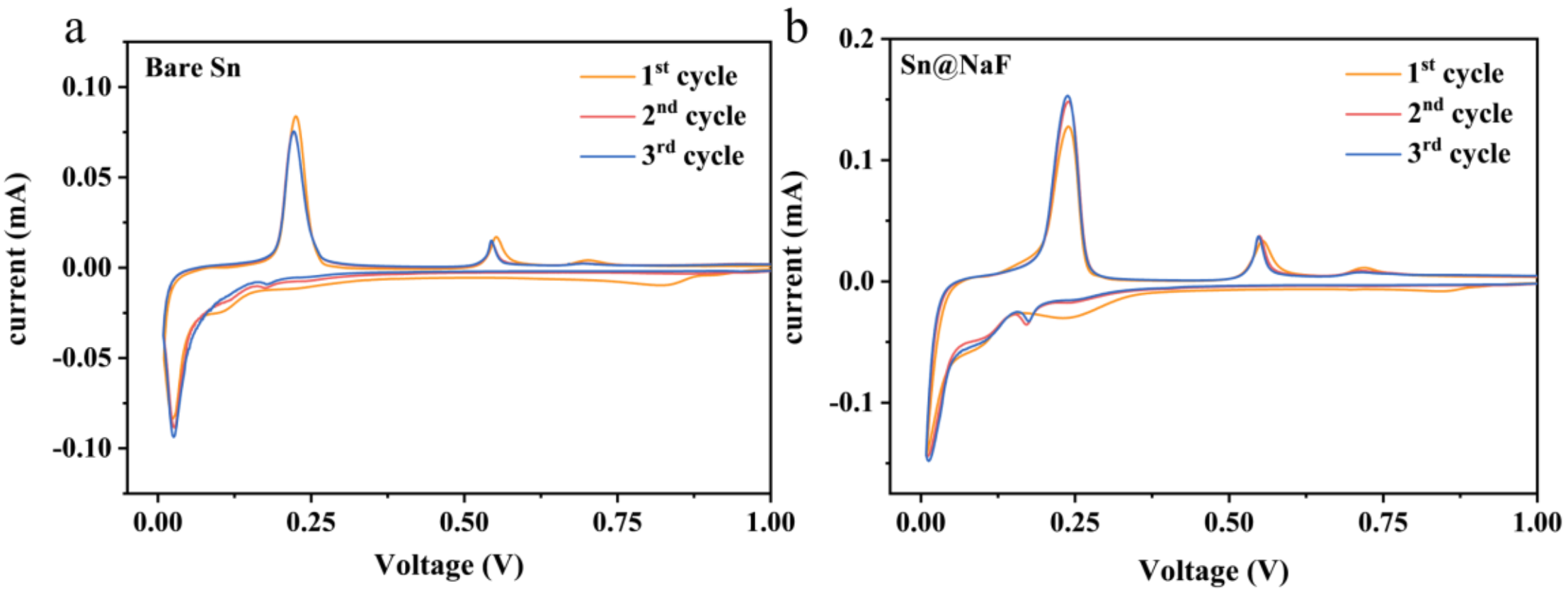


**Figure S10**. CV analysis of Bare Sn and Sn@NaF. The cyclic voltammetry curves of (a) Bare Sn and (b) Sn@NaF at 0.1 mV $s^{-1}$.

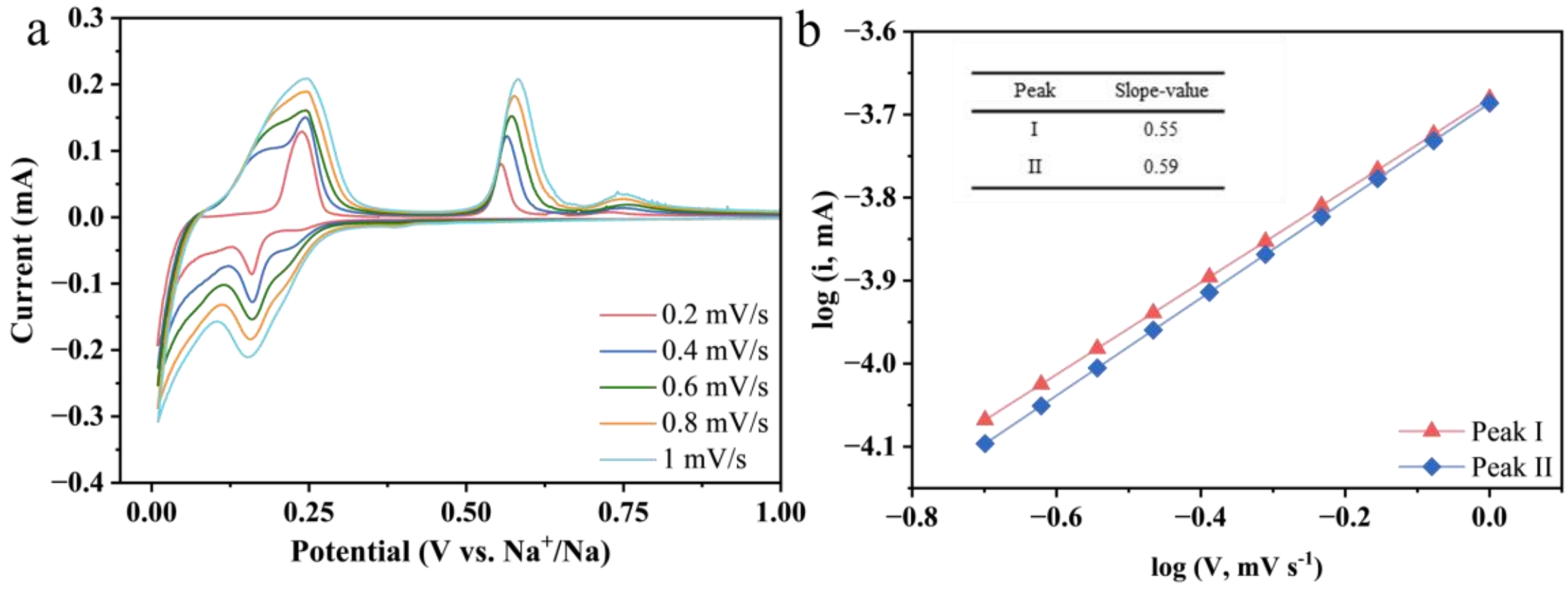


**Figure S11**. (a) CV curves of Sn@NaF ranging from 0.2 to 1 mV $s^{-1}$. (b) The calculated b values based on the logarithmic relationship between the peak current and scan rate.

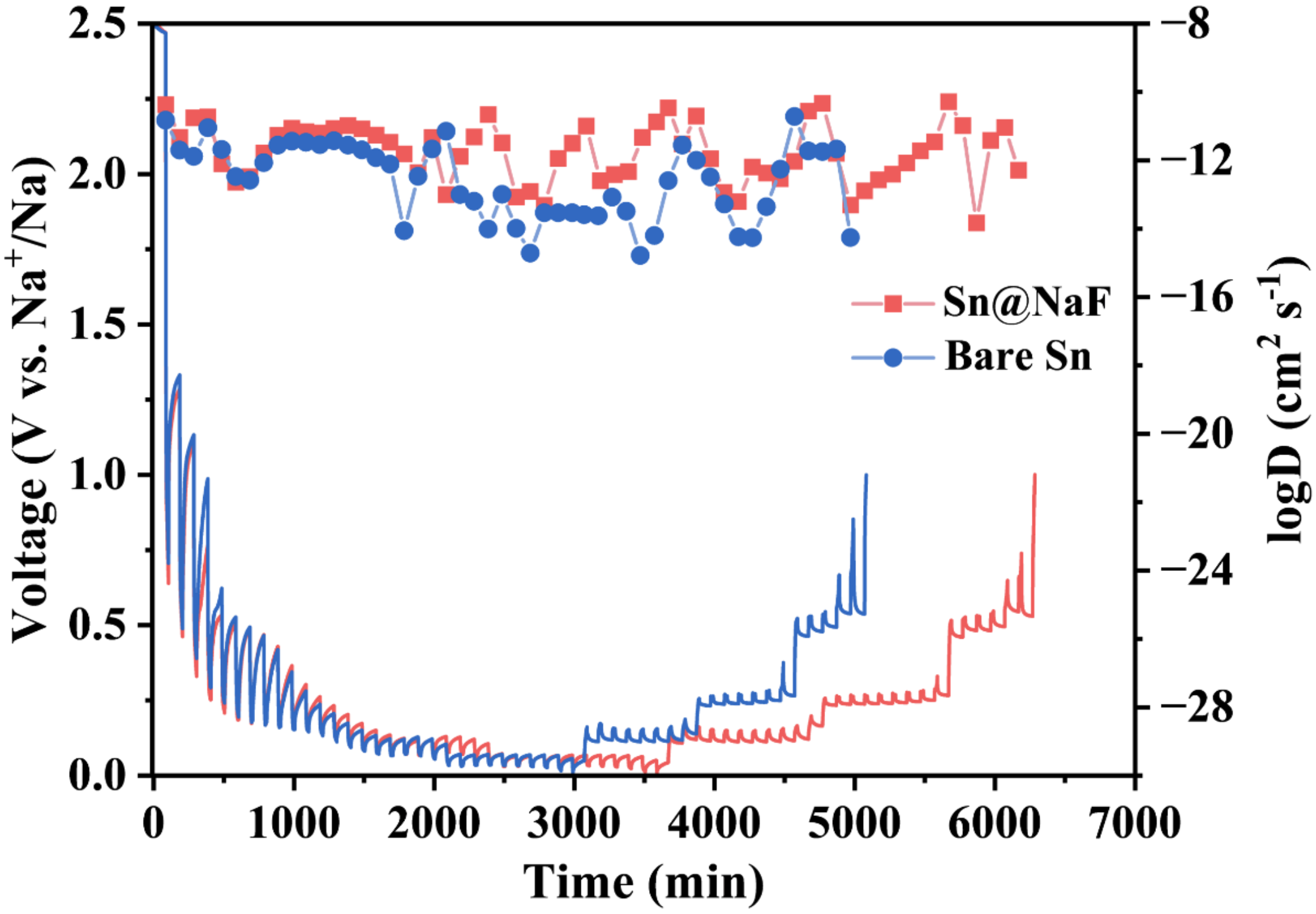


**Figure S12**. GITT curves and diffusion coefficients of the $Na^+$ ($D_{Na^+}$) of Bare Sn and Sn@NaF.

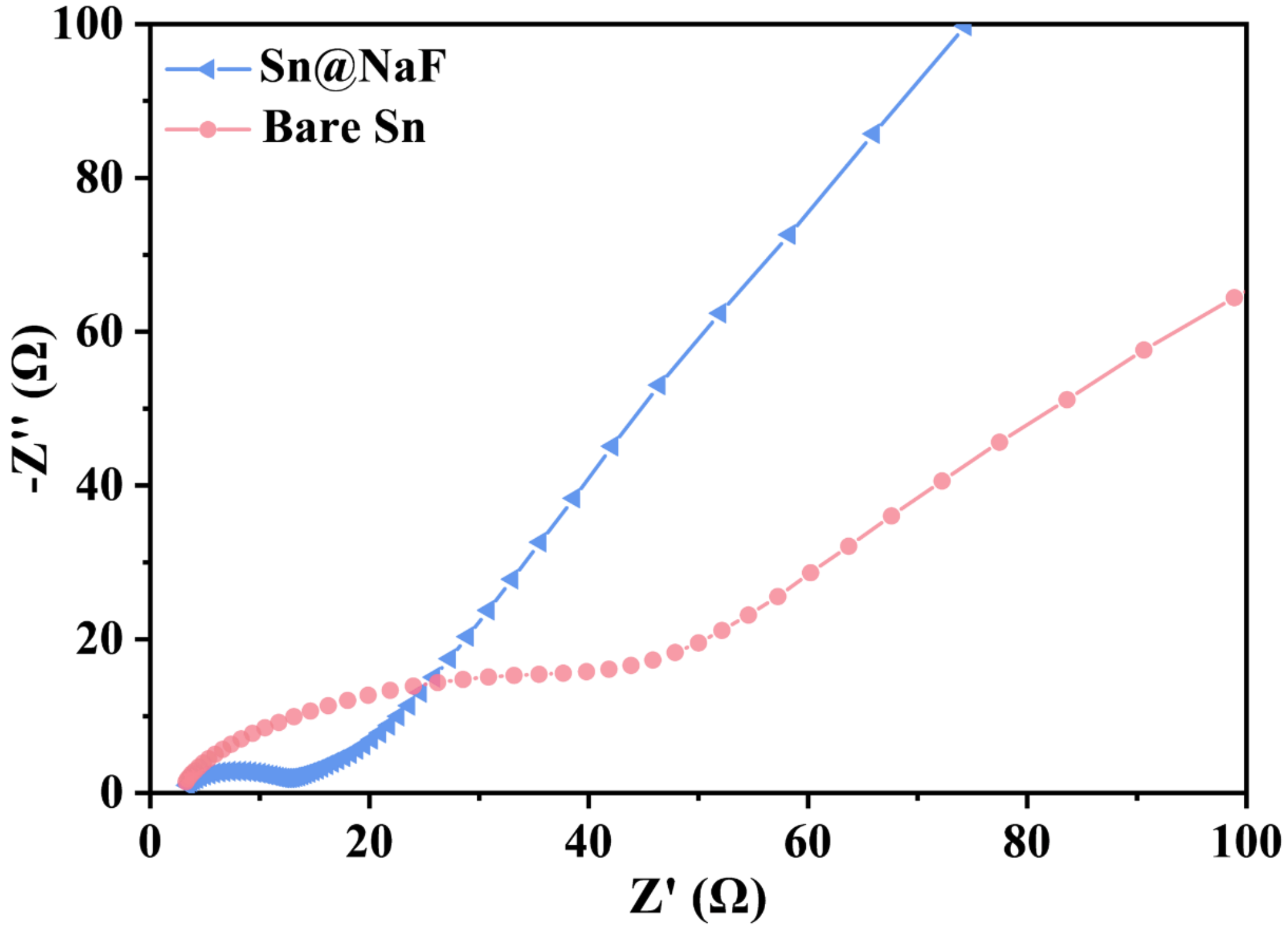


**Figure S13**. EIS of Bare Sn and Sn@NaF electrode after 5 cycles.

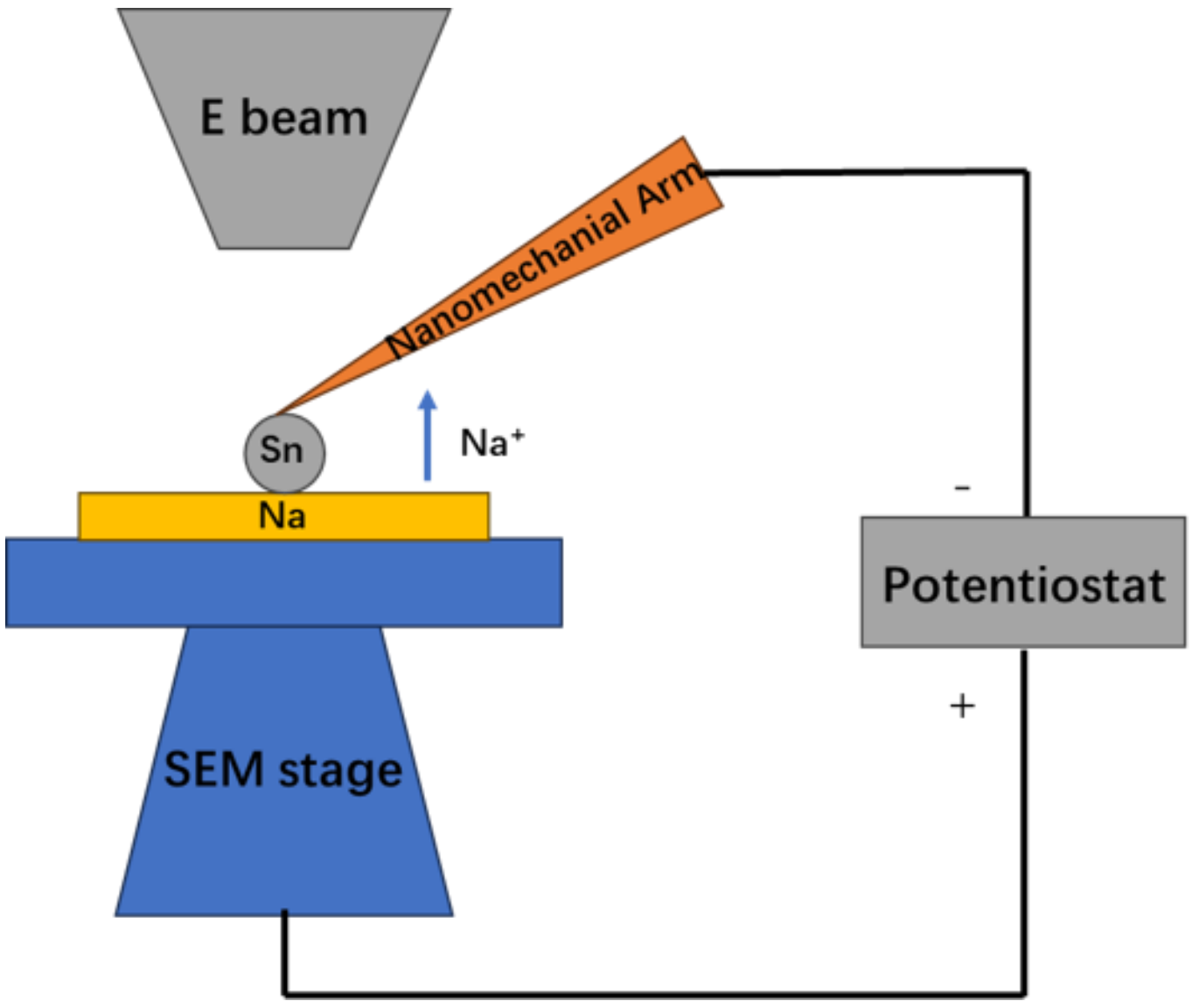


**Figure S14**. Working principle diagram of in-situ SEM.

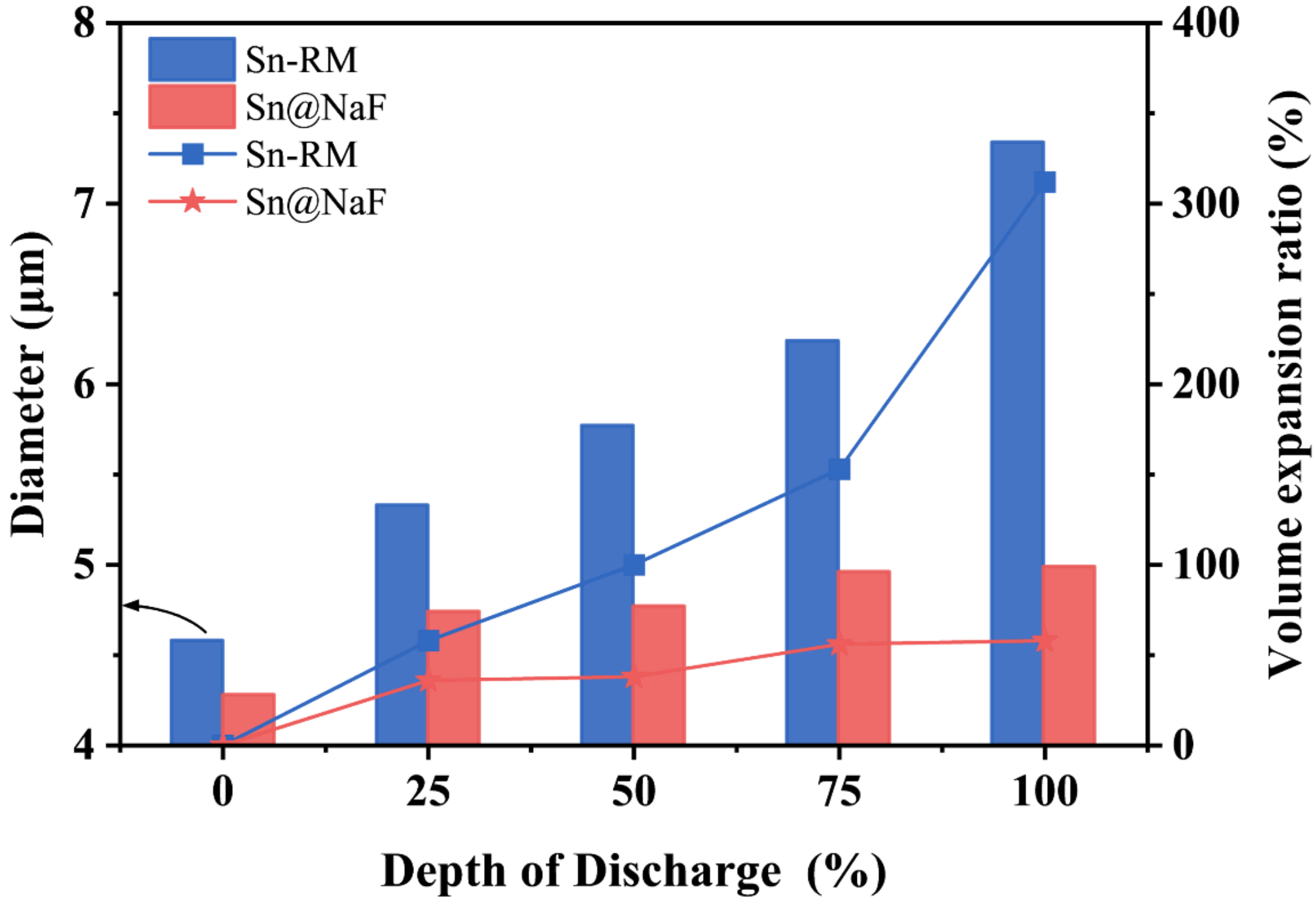


**Figure S15**. The comparation of the volume expansion rate and diameter change of Bare Sn and Sn@NaF at different discharge depths.

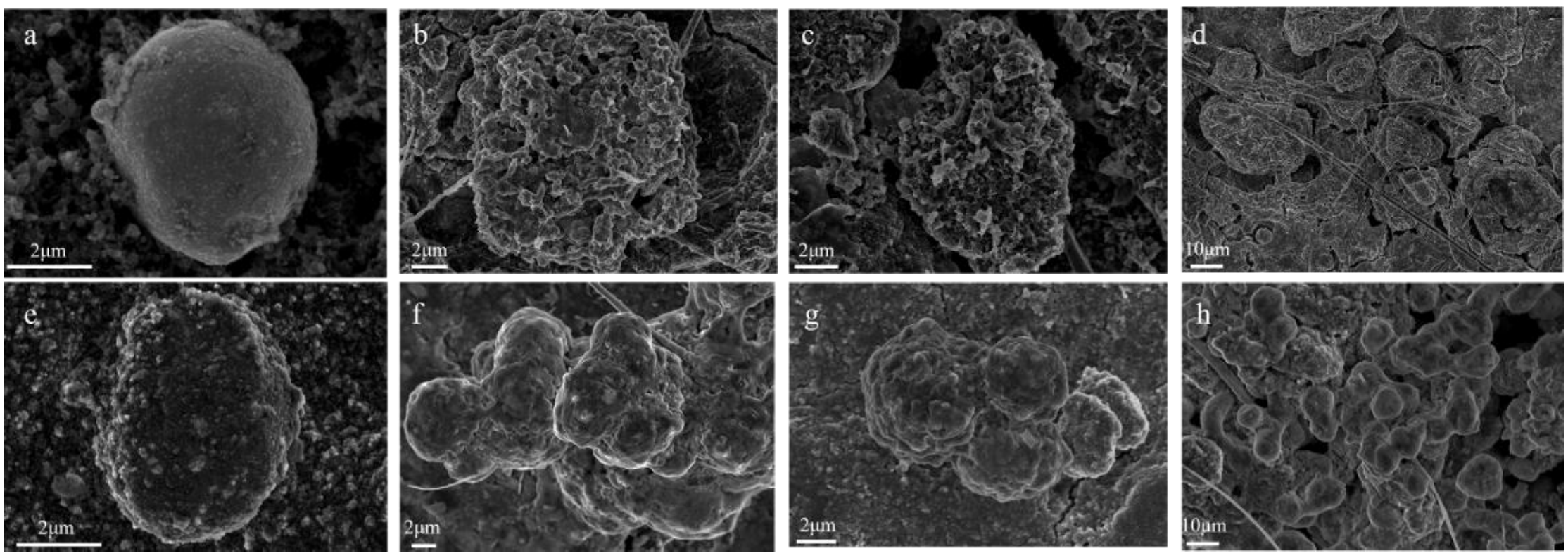

**Figure S16**. Ex-situ SEM images of Bare Sn and Sn@NaF plates during cycling. SEM image of the electrode of (a) Bare Sn and (e)Sn@NaF before cycling. (b) Bare Sn and (f)Sn@NaF after the first discharge. (c) Bare Sn and (g)Sn@NaF after the first charge. (d) Bare Sn and (h)Sn@NaF after 100 cycles of Sn@NaF.

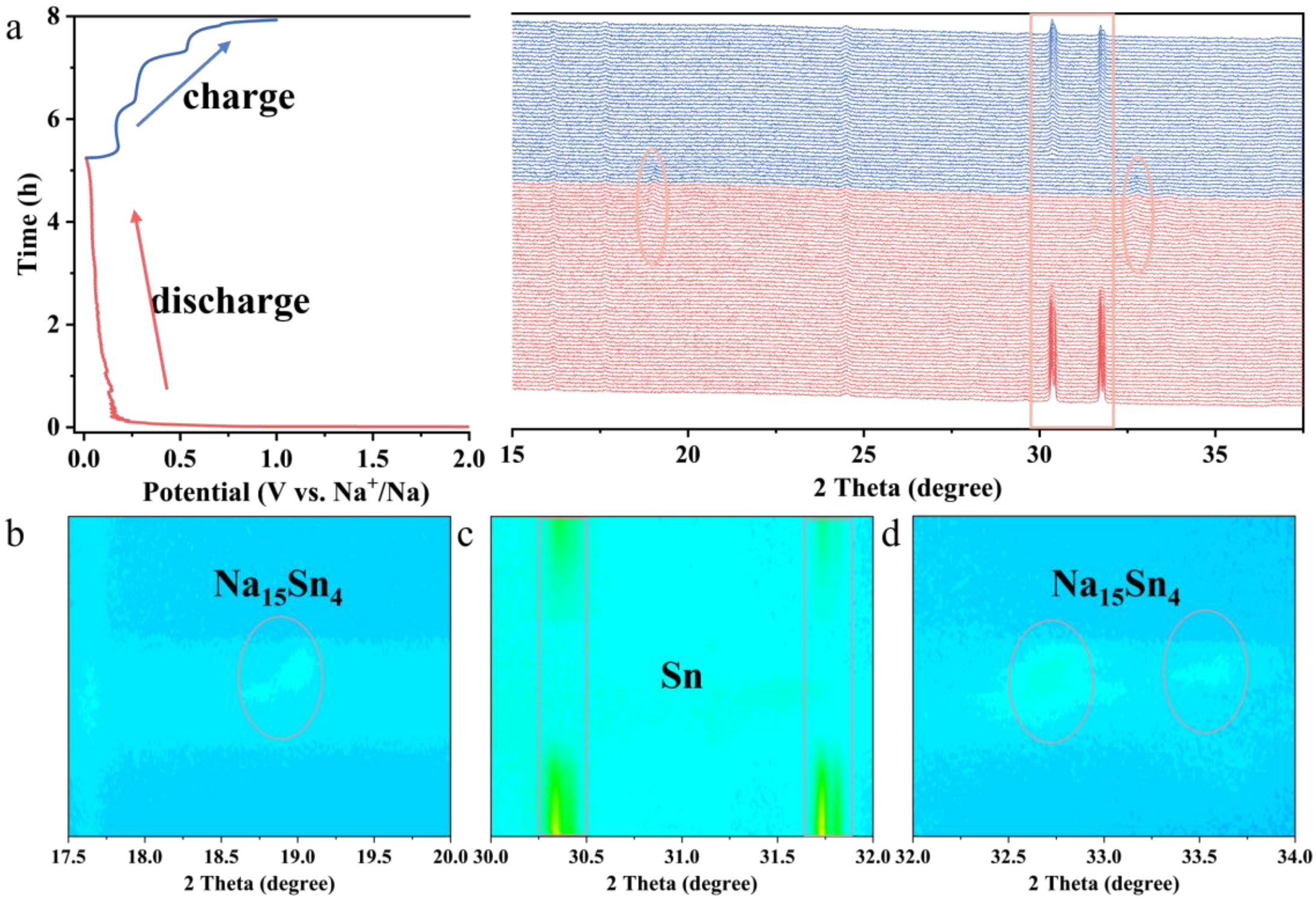


**Figure S17**. Electrochemical reactions of Sn@NaF in SIBs. (a) Galvanostatic discharge/charge profiles and the corresponding in-situ XRD patterns in the first cycle. (b-d) The contour maps of in-situ XRD.

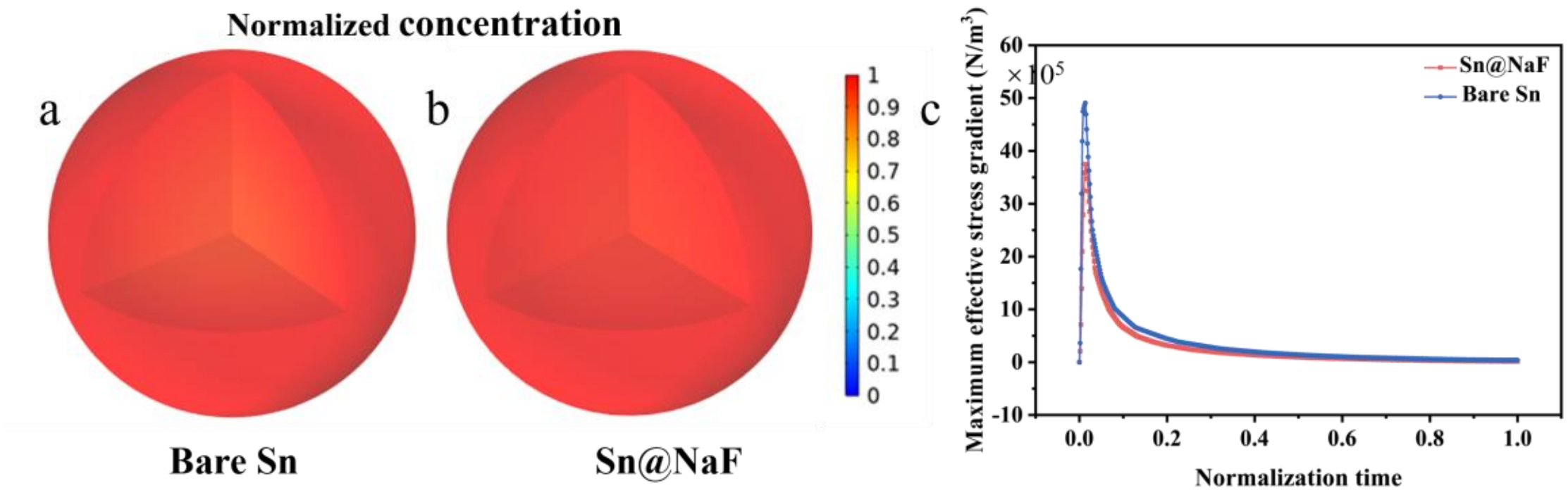


**Figure S18**. Chemo-mechanical simulated normalized $Na^+$ concentration and effective stress in the cross sections of the fully sodiated Bare Sn (a) and Sn@NaF (b). (c) Maximum effective stress gradient map of Bare Sn and Sn@NaF.

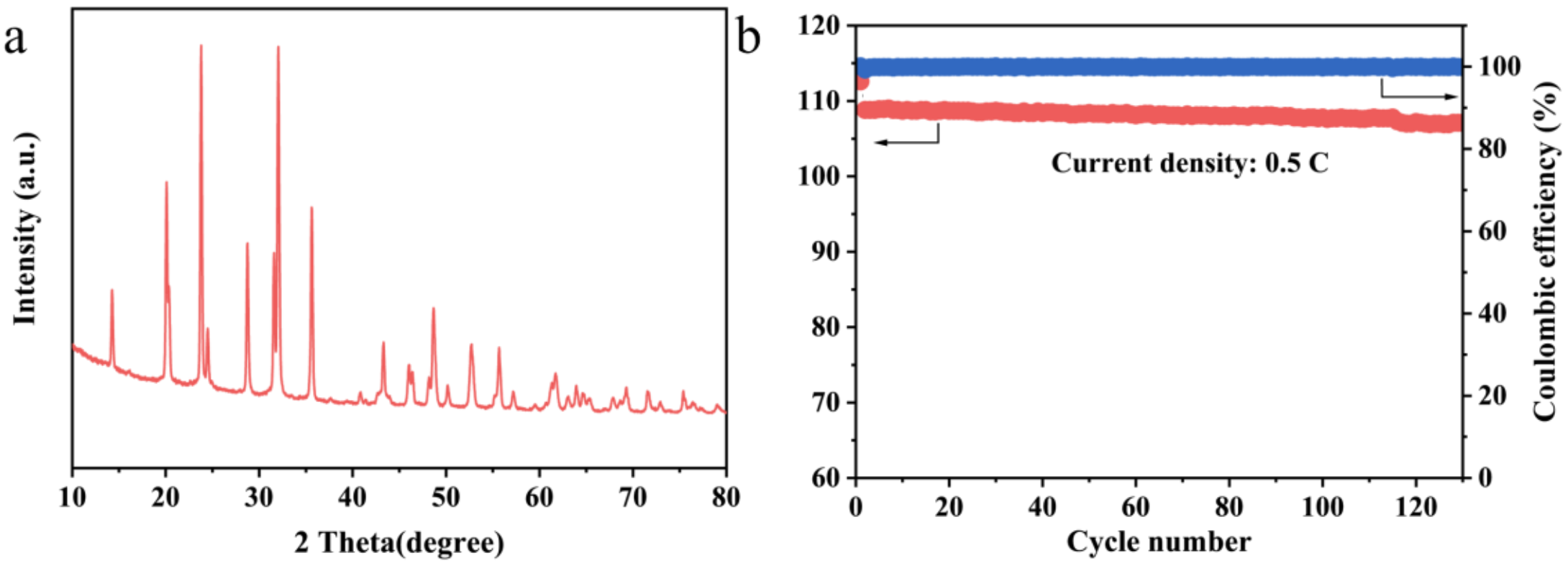


**Figure S19**. (a) XRD pattern of the synthesized NVP. (b) Cycling performance diagram of NVP.

REFERENCES

(1) Edison E, Sreejith S, Ren H *et al*. Microstructurally engineered nanocrystalline Fe–Sn–Sb anodes: towards stable high energy density sodium-ion batteries. *J Mater Chem A* 2019; **7**: 14145–14152.
(2) Edison E, Satish R, Ling WC *et al*. Nanostructured intermetallic $FeSn_2$-carbonaceous composites as highly stable anode for Na-ion batteries. *J Power Sources* 2017; **343**: 296–302.
(3) Chen SQ, Ao ZM, Sun B *et al.* Porous carbon nanocages encapsulated with tin nanoparticles for high performance sodium-ion batteries. *Energy Storage Mater* 2016; **5**: 180–190.
(4) Zhang ZX, Li SQ, Gao SL *et al*. Sn/P@G-CNTs composites as high-performance anode materials for sodium-ion batteries. *Electrochim Acta* 2021; **388**: 138532.
(5) Palaniselvam T, Goktas M, Anothumakkool B *et al*. Sodium storage and electrode dynamics of tin–carbon composite electrodes from bulk precursors for sodium-ion batteries. *Adv Funct Mater* 2019; **29**: 1900790.
(6) Liu Y, Liu X, Wang XY *et al*. Synergistic bimetallic effects of BiSb anodes enable long-stable sodium storage. *Adv Funct Mater* 2025; **35**: 2415092.
(7) Ying HJ, Zhang SL, Meng Z *et al*. Ultrasmall Sn nanodots embedded inside N-doped carbon microcages as high-performance lithium and sodium ion battery anodes. *J Mater Chem A* 2017, **5**, 8334–8342.
(8) Liu YC, Zhang N, Jiao LF *et al*. Ultrasmall Sn nanoparticles embedded in carbon as high-performance anode for sodium-ion batteries. *Adv Funct Mater* 2015; **25**: 214–220.
